# Automated biomarker candidate discovery in imaging mass spectrometry data through spatially localized Shapley additive explanations

**DOI:** 10.1016/j.aca.2021.338522

**Published:** 2021-04-26

**Authors:** Leonoor E.M. Tideman, Lukasz G. Migas, Katerina V. Djambazova, Nathan Heath Patterson, Richard M. Caprioli, Jeffrey M. Spraggins, Raf Van de Plas

**Affiliations:** a Delft Center for Systems and Control, Delft University of Technology, Delft, Netherlands; b Mass Spectrometry Research Center, Vanderbilt University, Nashville, TN, USA; c Department of Biochemistry, Vanderbilt University, Nashville, TN, USA; d Department of Chemistry, Vanderbilt University, Nashville, TN, USA; e Department of Pharmacology, Vanderbilt University, Nashville, TN, USA; f Department of Medicine, Vanderbilt University, Nashville, TN, USA

**Keywords:** Supervised machine learning, Model interpretability, Biomarker discovery, Imaging mass spectrometry, Shapley additive explanations, Explainable artificial intelligence

## Abstract

The search for molecular species that are differentially expressed between biological states is an important step towards discovering promising biomarker candidates. In imaging mass spectrometry (IMS), performing this search manually is often impractical due to the large size and high-dimensionality of IMS datasets. Instead, we propose an interpretable machine learning workflow that automatically identifies biomarker candidates by their mass-to-charge ratios, and that quantitatively estimates their relevance to recognizing a given biological class using Shapley additive explanations (SHAP). The task of biomarker candidate discovery is translated into a feature ranking problem: given a classification model that assigns pixels to different biological classes on the basis of their mass spectra, the molecular species that the model uses as features are ranked in descending order of relative predictive importance such that the top-ranking features have a higher likelihood of being useful biomarkers. Besides providing the user with an experiment-wide measure of a molecular species’ biomarker potential, our workflow delivers spatially localized explanations of the classification model’s decision-making process in the form of a novel representation called SHAP maps. SHAP maps deliver insight into the spatial specificity of biomarker candidates by highlighting in which regions of the tissue sample each feature provides discriminative information and in which regions it does not. SHAP maps also enable one to determine whether the relationship between a biomarker candidate and a biological state of interest is correlative or anticorrelative. Our automated approach to estimating a molecular species’ potential for characterizing a user-provided biological class, combined with the untargeted and multiplexed nature of IMS, allows for the rapid screening of thousands of molecular species and the obtention of a broader biomarker candidate shortlist than would be possible through targeted manual assessment. Our biomarker candidate discovery workflow is demonstrated on mouse-pup and rat kidney case studies.

## Graphical Abstract

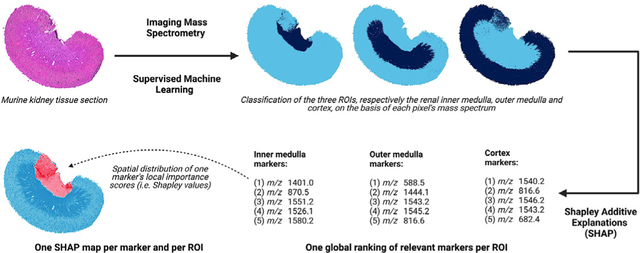

## 1. Introduction

A biomarker can generally be considered an objectively measurable indicator of a specific biological state or disease condition [1,2]. Biomarkers can be used to differentiate between anatomical structures, cell types, and disease states, and lend themselves to the screening, diagnosis, and monitoring of disease, the identification of new drug targets, and the assessment of therapeutic response [1,3–5]. In our work, the term “biomarker candidate” refers to a putative molecular biomarker (i.e. a chemical species) that is differentially expressed between biological states [2]. One technology for discovering such molecular markers at scale is mass spectrometry, which characterizes molecular species in terms of their mass-to-charge ratio (*m/z*). Imaging mass spectrometry (IMS) is a multiplexed, label-free imaging technology that uses mass spectrometry for the molecular mapping of tissues down to cellular resolution [6–8]. An IMS experiment involves collecting spatially localized mass spectra for each pixel in a grid of measurement locations across a sample surface [9,10]. Each pixel has an associated mass spectrum and each mass spectrum plots the measured signal intensity, which is indicative of relative abundance, versus the analytes’ *m/z* values. The spatial distribution and relative abundance of an analyte can be visualized as an ion image, which plots the signal intensity measured for that analyte across all pixels of the sample’s surface [11,12]. IMS is an excellent tool for biomarker discovery for the following three reasons: it is able to concurrently detect hundreds to thousands of analytes within a single experiment in an untargeted manner, it can probe analytes from a wide range of molecular classes (e.g. peptides, proteins, lipids, glycans, metabolites), and it enables the mapping of analytes’ spatial distributions in relation to the (patho)histology of tissue samples [5,13,14].

One way novel biomarker candidates can be discovered is by observing the differential expression of molecules between distinct sample classes (e.g. different cell types, different organs, different stages of a disease) [2,15]. However, the large size and high-dimensionality of IMS datasets, which commonly yield several hundreds of thousands of pixels and several hundreds to thousands of molecular ions tracked per pixel, pose a challenge. Manually examining the spatial mapping of thousands of molecular species across the surface of a sample is laborious and risks introducing human subjectivity into the process, leading to results whose reproducibility cannot necessarily be guaranteed [11,16]. The amount of data generated by IMS experiments is so large that it has become more efficient (and in many cases necessary) to computationally search for biomarker candidates among a multitude of ion intensity signals [5,17]. In this work, we suggest a machine learning (ML) workflow for performing biomarker candidate discovery that provides one with a shortlist of molecular species that are characteristic of the class for which biomarkers are sought. Our approach uses supervised ML models to classify mass spectra into different biological classes of interest and then uses state-of-the-art methods from the field of interpretable ML [18–20] to determine the discriminative relevance, and biomarker potential, of each molecular species.

**Fig. 1.**
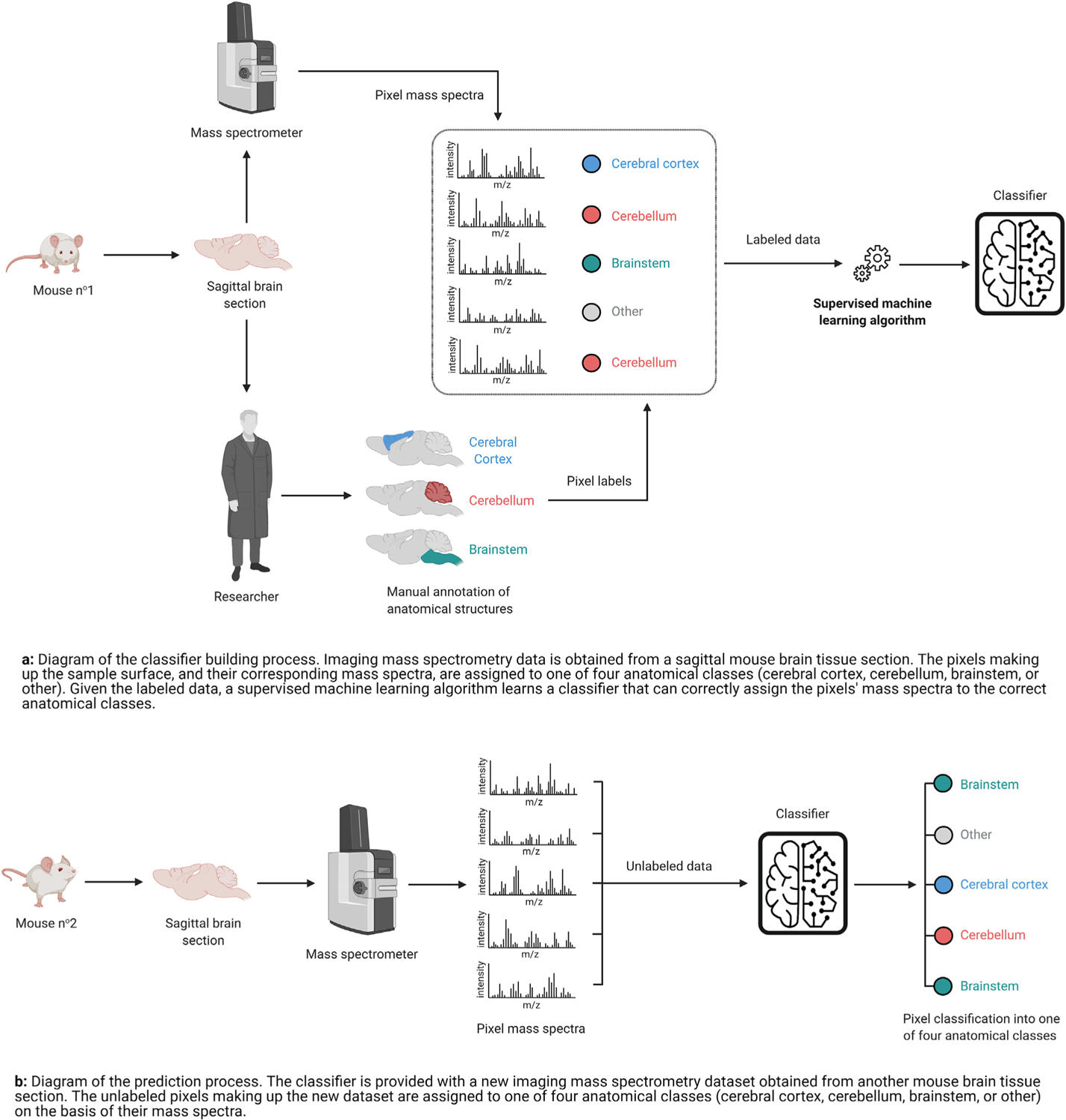
Diagrams of the classifier building and prediction processes in imaging mass spectrometry. Icons from Refs. [21,22].

In our work, an IMS dataset is represented by a data matrix $X\in {\mathbb{R}}^{m\times n}$ whose rows *x*_*i*_ = *X*_(*i*,:)_, for *i* = 1, 2, 3…*m*, correspond to the mass spectra of the pixels making up the sample’s surface and whose columns *x*^j^ = *X*_(:,j)_, for *j* = 1; 2; 3…*n*, correspond to the *m/z* bins per spectrum. The *m* rows and *n* columns of *X* can be respectively referred to as observations and features. Classification is a form of supervised ML in which the observations *x*_*i*_ are annotated with discrete class labels *y*_*i*_ that represent user-provided knowledge related to these observations. Binary classification problems involve a positive class (e.g. diseased tissue), labeled as *y*_*i*_ = +1; and a negative class (e.g. healthy tissue), labeled as *y*_i_ = − 1. The positive class is usually the class of interest: in our case studies, it is the class for which we want to discover biomarker candidates. Problems with multiple target classes (e.g. multiple cell types or functional tissue units) can be decomposed into multiple binary classification problems, each of which involve differentiating one class from the remaining classes. In the context of our work, classification is therefore the task of learning a multivariate function $f^{*}:{\mathbb{R}}^{n}\mapsto \left\{-1,+1\right\}$, called a classifier or classification model, that assigns each pixel to a class according to the molecular information provided by its mass spectrum *x*_*i*_. Note a difference between the classifier’s discrete class prediction ${\hat{y}}_{i}=f^{*}\left(x_{i}\right)\in \left\{-1,+1\right\}$ and its continuous raw output $f\left(x_{i}\right)\in \mathbb{R}$, where $f:{\mathbb{R}}^{n}\mapsto \mathbb{R}$. The classifier’s prediction is the class label assigned to a particular observation *x*_*i*_, whereas the classifier’s raw output can be interpreted as the score (e.g. probability, log-odds ratio) of *x*_*i*_ being assigned to the positive class. Fig. 1a illustrates the process of building a classifier in IMS: a supervised ML algorithm fits a classification model to a labeled IMS dataset called the training dataset (i.e. mass spectra *x*_*i*_ whose class membership *y*_*i*_ is known). The resulting model can then be used to classify new unlabeled data (i.e. mass spectra *x*_*i*_ whose class membership *y*_*i*_ is unknown) as illustrated by Fig. 1b. The performance of a classifier is measured by its ability to generalize, that is to correctly predict the labels for new data instances such that ${\hat{y}}_{i}=y_{i}$.

Traditionally, applications of supervised ML in IMS focus on maximizing the predictive performance of classifiers designed to automate user-defined recognition tasks, without necessarily examining their decision-making processes. However, we suggest that examining the relationship between a classifier’s features and its prediction is important because it can reveal which features, and thus which molecular species, enable the differentiation of classes.

Model interpretability is the ability to explain the predictions of a supervised ML model by reporting the relative predictive importance of its features^1^. The importance, or relevance, of a feature is a measure of how it influences the model’s prediction, considering both its direct effect (i.e. statistical association with the prediction) and its indirect effect (i.e. statistical association between features) [19,26]. The local predictive importance of a feature measures its influence on the predictive model’s output for a specific observation (e.g. the mass spectrum of one pixel), whereas the global predictive importance of a feature measures its influence on the predictive model’s output for all observations (e.g. all pixels of a sample) [19,20,26]. In addition to reporting which features drive the decision-making processes of supervised ML models, interpretability methods also facilitate model troubleshooting (e.g. debugging, monitoring, checking for bias). For example, in the context of IMS data analysis, interpretability methods make it possible to trace whether the decision-making process of a classifier is based on genuine biological patterns rather than on instrumental patterns or chemical noise that are spuriously associated to the class labels. ML interpretability methods effectively address the issue of supervised ML algorithms producing “black-box” models with unintelligible predictive mechanisms [18–20]. The importance of ML interpretability for knowledge discovery has recently been discussed in genomics [27] and single-cell mass spectrometry [28]. To our knowledge, our work is the first application of ML interpretability methods to IMS data for the purpose of biomarker candidate discovery. Our aim is to formulate and demonstrate how ML interpretability methods can be used to understand how the spatial distribution and relative abundance of certain molecular species relate to the classification of different regions of a tissue sample, effectively automating biomarker candidate discovery in IMS data.

Our approach to aiding biomarker discovery is to automate and accelerate the identification of discriminative features by empirically learning which molecular species’ overexpression or underexpression enable the recognition of a user-defined class [15]. We translate the problem of biomarker discovery into a feature ranking problem: ML interpretability methods computationally estimate the importance of each feature, or *m/z* value, with regards to a specific classification task and produce a ranking of the features in descending order of predictive importance. Ranking the features in terms of predictive importance facilitates the identification of a shortlist of molecular species that are characteristic of a class of interest, and thus have a higher likelihood of being useful biomarkers. In addition to providing one with a global understanding of which molecular species hold potential for recognizing a user-provided class, our approach uses SHAP maps to give the user spatially localized insight into each biomarker candidate’s relationship with the class of interest. SHAP maps are a novel graphical representation of a model’s decision-making process that can yield a nuanced local assessment of a biomarker candidate’s potential and spatial specificity. Our biomarker candidate discovery workflow is therefore a scalable computational tool that enables one to rapidly, efficiently, and automatically filter the multitude of molecular species recorded by IMS down to a panel of promising biomarker candidates that deserve further study and validation.

## 2. Machine learning methodology

### 2.1. Extreme gradient boosting for imaging mass spectrometry data classification

There are many applications of supervised ML in IMS: random forests [17,29,30], support vector machines [29,31], convolutional neural networks [32,33], and gradient boosting machines [34,35] are frequently used classification model types. Decision trees are particularly suitable for IMS data analysis because they are non-linear and non-parametric predictive models that can account for complex dependencies between features, do not make assumptions about the underlying data distribution, and do not require feature scaling. A decision tree is a directed graph that partitions the feature space by recursive binary splitting: its nodes correspond to subsets of the data, and its branches correspond to the partitioning of a feature above or below a splitting threshold [36–38]. Given that a single decision tree is neither flexible nor stable enough to achieve high predictive performance on IMS data classification tasks, combining multiple decision trees into an ensemble model is usually a preferable strategy [37,39]. We therefore choose to use XGBoost models for classification. XGBoost is a fast and scalable implementation of (stochastic) regularized gradient boosting that was developed by Chen and Guestrin in 2016 [40] based on the work of Friedman [41,42], Freund and Schapire [43]. An XGBoost model is an ensemble of regression trees (i.e. decision trees that output real values in their terminal nodes) that can perform classification by additive logistic modeling [44,45].


(1) $$\underset{f}{\text{min}}\overset{m_{\text{train }}}{\underset{i=1}{\sum }}ℒ\left(y_{i},{\hat{y}}_{i}\right)+\overset{K}{\underset{k=1}{\sum }}\Omega \left(\tau_{k}\right)$$

Regularized gradient boosting is a forward stagewise additive modeling algorithm for solving numerical optimization problems of the form of Equation (1). ℒ is a differentiable loss function (e.g. negative log-likelihood) that measures the difference between the observations’ labels *y*_*i*_ and the predictive model’s predictions ${\hat{y}}_{i}=f^{*}\left(x_{i}\right)$, and Ω is a regularization term that penalizes the complexity of the regression trees making up the ensemble in order to avoid overfitting^2^ [40,46]. In Equation (1), the regression trees are written *τ*_*k*_, for *k* = 1; 2; 3…*K*; and *m*_*train*_ refers to the number of observations making up the training dataset. The XGBoost algorithm builds a classification model from sequentially added regression trees, each of which is focused on the observations that the previously added trees classified incorrectly [46–48]. Given an initial prediction *τ*_0_ (e.g. the logarithm of the odds), the accuracy of the ensemble model is iteratively improved by functional gradient descent: each newly added regression tree is parameterized to approximate the negative gradient of the loss function ℒ [46]. In order to avoid overfitting, the contribution of each newly added regression tree is weighted using a shrinkage parameter *ν*, with 0 < *ν* < 1 (*ν* = 0.3 in our case studies), which determines the learning rate of the boosting procedure [40,41,49]. In our automated biomarker candidate discovery workflow, the XGBoost learning process is stochastic because the regression trees making up the ensemble are learned on randomized subsamples of the training set, and because the features used for node splitting are chosen among a random subset of features [40,49]. The idea is to randomly subsample the rows and columns of the data matrix during training to make each regression tree slightly different from the other regression trees, and hence prevent overfitting.


(2) $$f\left(x_{i}\right)=\text{log}\left(\frac{p\left(x_{i}\right)}{1-p\left(x_{i}\right)}\right)=\tau_{0}\left(x_{i}\right)+\overset{\text{K}}{\underset{\text{k}=1}{\sum }}v\tau_{\text{k}}\left(x_{i}\right)\;\text{with }p\left(x_{i}\right)=\text{Pr}\left(y_{i}=+1|x_{i}\right)$$

Equation (2) defines the raw output *f* (*x*_*i*_), or raw untransformed margin value, of an XGBoost classifier as the (natural) logarithm of the odds, called the log-odds [49,50]. The odds are defined as the ratio of the probability *p*(*x*_*i*_) of observation *x*_*i*_ being assigned to the positive class over the probability of observation *x*_*i*_ being assigned to the negative class. The XGBoost classifier is an additive logistic regression model because it represents the log-odds as a linear combination of regression trees [45,47]. The probability of the model predicting a positive outcome (i.e. assigning an observation to the class of interest) can be obtained from the log-odds thanks to a logistic transformation [45]: *p*(*x*_*i*_) = *S*(*f* (*x*_*i*_)) where S:ℝ↦[0,1] is the sigmoid function. Since the sigmoid function is a non-decreasing saturation function, an increase in the log-odds implies an increase in the probability of predicting a positive outcome, and, conversely, a decrease in the log-odds implies a decrease in the probability of predicting a positive outcome. The classification model’s prediction ${\hat{y}}_{i}=f^{*}\left(x_{i}\right)$ is either +1 and − 1 depending on whether *p* (*x*_*i*_) is above or below a given threshold η, with 0 < η < 1(η = 0.5 in our case studies).

### 2.2. Shapley additive explanations for measuring biomarker candidate relevance

Our workflow for biomarker candidate discovery in IMS data uses Shapley additive explanations (SHAP) to quantify the local and global predictive importance of features (e.g. *m/z* values in IMS) with respect to a given classification task. SHAP is a state-of-the-art interpretability method based on Shapley values from cooperative game theory. It regards the features as players that form coalitions (i.e. ordered subsets) to achieve the classification or regression model’s output, which is the game’s payout. SHAP is called a model-agnostic interpretability method because it can derive post-hoc explanations for the predictions of any type of classification model by relating its input to its outputs [19,20,26]. SHAP was developed by Lundberg and Lee [51,52] based on the work of Strumbelj and Kononenko [53,54], and on Ribeiro et al.’s idea of locally approximating the decision-making process of a “black-box” supervised ML model using inherently interpretable local surrogate models [55].

In order to explain the prediction made by a model on a specific observation, SHAP computes the contribution of each feature to the model’s output using Shapley values. The Shapley value of a feature is its contribution to the model’s output for a specific observation, averaged over all possible feature orderings [52,56]. In the words of Lundberg et al., “Shapley values are computed by introducing each feature, one at a time, into a conditional expectation function of the model’s output, and attributing the change produced at each step to the feature that was introduced” [56]. Equation (3) defines the Shapley value $\varphi_{i}^{j}\left(f,x_{i}\right)$ of feature *x*^*j*^, with *j* ∈ {1, 2, 3…*n*}, when explaining the predictive model’s decision-making process for one specific observation *x*_i_ = *X*_(*i*,:)_, with *i*∈{1, 2, 3…*m*}. Since a feature’s contribution to the model’s output depends on the order in which other features were introduced, the feature’s Shapley value is obtained by averaging its contribution over all possible feature orderings. In Equation (3), the set of all possible feature orderings is written *Π*. The set of features that we are conditioning on, written $S_{j}^{\pi }$, is the set of all features that precede feature *x*^*j*^ in ordering π.


(3) $$\phi_{i}^{j}\left(f,x_{i}\right)=\underset{\pi \in \Pi }{\sum }\frac{1}{n!}\left[f_{x_{i}}\left(S_{j}^{\pi }\cup j\right)-f_{x_{i}}\left(S_{j}^{\pi }\right)\right]$$

The set function $f_{x_{i}}\left(S_{j}^{\pi }\right)$ is defined by Equation (4) as the conditional expectation of the model’s output. The *n*-dimensional vector *x*_i_ is considered to be a random variable where only the features belonging to subset $S_{j}^{\pi }$ (i.e. the features before *x*^*j*^ in the feature ordering π) are known [57]. The unknown features (i.e. the features after *x*^*j*^ in the feature ordering p) are obtained by sampling from the training dataset [57]. In Equation (4), *f* (*x*_*i*_) is the model’s raw output for observation *x*_*i*_, rather than the predicted class label *f* *(*x*_*i*_)∈ { − 1, + 1}. SHAP does not require knowledge of an observation’s true class label (*y*_*i*_ for *i*∈1, 2, 3…*m*) to evaluate the degree to which a model depends on a specific feature. SHAP can therefore be used to explain the decision-making process of a model on new unlabeled data, which is useful for measuring the influence of each feature on the model’s generalization performance.


(4) $$f_{x_{i}}\left(S_{j}^{\pi }\right)=E\left[f\left(x_{i}\right)|S_{j}^{\tau }\right]$$

The sign of a feature’s Shapley value provides information about the direction of its effect on a predictive model’s output. A positive Shapley value indicates that feature *x*^*j*^ increases the raw output *f* (*x*_*i*_) of the predictive model for observation *x*_*i*_. Conversely, a negative Shapley value indicates that feature *x*^*j*^ decreases the raw output. The Shapley value’s magnitude indicates how strongly the corresponding feature influences the model’s local decision-making process. In our work, we refer to the Shapley value of a feature for a given observation as its local SHAP importance score. In the context of IMS data classification, the Shapley value $\varphi_{i}^{j}\left(f,x_{i}\right)$ reports the contribution of the *j*^th^ *m/z* bin or peak when assigning the *i*^th^ pixel’s mass spectrum to a class. Computing the local SHAP importance scores of all features (i.e. *m/z* values) for all observations (i.e. mass spectra) yields an *m* × *n* matrix whose (*i*,*j*)^th^ entry is the Shapley value of feature *x*^*j*^ for observation *x*_*i*_.

SHAP owes its reliability to the fact that it satisfies the local accuracy and consistency properties [56]. The local accuracy property, also known as the efficiency property in cooperative game theory, guarantees that the Shapley values of all features add up to the difference between the predictive model’s raw output *f* (*x*_*i*_) for a given observation *x*_*i*_ and the model’s expected output *E*[*f* (*x*_*i*_)] over the entire dataset [56]. The local accuracy property is given by Equation (5). SHAP offers contrastive explanations that compare the model’s local output to its average global output. In IMS terminology, the local accuracy property states that, given a mass spectrum of interest, the sum of the Shapley values of its molecular features (i.e. *m/z* values) is equal to the classifier’s raw output for that mass spectrum minus the classifier’s average raw output over all mass spectra. SHAP distributes the difference between the classifier’s output for a mass spectrum of interest and the classifier’s average output, among the different *m/z* values that the classifier uses as inputs.


(5) $$f\left(x_{i}\right)=\varphi_{0}(f)+\overset{n}{\underset{j=1}{\sum }}\varphi_{i}^{j}\left(f,x_{i}\right)\text{ with }\varphi_{0}(f)=E\left[f\left(x_{i}\right)\right]$$

The consistency property, also known as the monotonicity property in cooperative game theory, states that if a model changes so that some feature’s influence on the output increases, the importance score assigned to that feature does not decrease [58]. Consistency is necessary for the ranking of a model’s features according to their importance scores because it guarantees that a feature with a higher importance score than another feature is actually more important to the model than the other feature. Note that impurity-based measures of global feature importance, which are popular for measuring feature importance in decision tree ensembles^3^ and have been used in IMS [34], are actually inconsistent and can therefore produce unreliable feature rankings [58].


(6) $$\Phi_{j}(f,X)=\frac{1}{m}\overset{m}{\underset{i=1}{\sum }}\left|\varphi_{i}^{j}\left(f,x_{i}\right)\right|$$

A global measure of feature importance can be obtained by averaging the magnitude of each feature’s local SHAP scores, or Shapley values, over all observations in the dataset [56]. Equation (6) defines what we refer to as the global SHAP score Φ_*j*_ of feature *x*^*j*^ = *X*_(:,*j*)_ for *j*∈{1, 2, 3…*n*}. The global SHAP score of a feature quantifies its influence on the model’s decision-making process, averaged over all possible feature orderings and all observations. Computing the global SHAP importance scores of all features yields an *n*-dimensional vector where *n* is the total number of features. In the context of IMS data analysis, the global SHAP score of a feature is an experiment-wide measure of the feature’s predictive importance with respect to a given classification task. Promising biomarker candidates can be easily identified by ranking IMS features (i.e. *m/z* values) in descending order of global SHAP importance. Retaining the top-ranking features yields a shortlist of biomarker candidates that are worthy of further study.

In our workflow for biomarker candidate discovery in IMS data, we use a fast implementation of SHAP called TreeSHAP, or TreeExplainer [62], that is specific to decision tree based predictive models like XGBoost. Unlike other SHAP implementations (e.g. KernelSHAP) that calculate sampling-based approximations of Shapley values (often in exponential time), TreeSHAP is able to compute the exact Shapley values of features within low-order polynomial time by exploiting the structure of decision trees [56,58]. When using TreeSHAP to measure the local and global SHAP importance scores of features, one has to choose between two feature perturbation approaches [62]. In this paper, we opt for the tree-path dependent approach because it involves computing the observational, rather than interventional, Shapley values [57]. Observational Shapley values are defined by Equations (3) and (4), whereas interventional Shapley values define the set function differently. The difference between observational and interventional Shapley values relates to how SHAP handles statistical dependencies between the features that the model uses as inputs [57]. Accounting for high-dimensional feature dependencies is what makes measuring the predictive importance of IMS features, many of which are involved in common biochemical processes, particularly challenging. Another measure of global feature predictive importance, called permutation importance (PI), has been used for ranking IMS features with regards to tissue classification tasks [17] despite it only partially accounting for feature inter-dependencies. PI defines the importance of a feature as the average decrease in model accuracy when its values are randomly permuted across all observations^4^. The feature importance scores delivered by PI can be misleading when the classifier’s features exhibit statistical dependencies [65]. Unlike PI, SHAP accounts for linear and non-linear feature inter-dependencies [56]. Furthermore, PI relies upon out-of-distribution data instances that are not necessarily realistic [66], whereas computing the global SHAP score of a feature using observational Shapley values constrains the sampling of unknown features to a range of values (i.e. partitions of the feature space) allowed by the decision trees making up the ensemble [64]. A detailed discussion of how TreeSHAP computes observational Shapley values, and how observational Shapley values handle feature dependencies, is beyond the scope of this paper, and we therefore refer the reader to Refs. [56–58]. Observational Shapley values are recommended for knowledge discovery in biology and chemistry because they spread credit among correlated features that are jointly informative of the outcome of interest [57].

### 2.3. SHAP maps for a spatial understanding of a classifier’s decision-making process

**Fig. 2.**
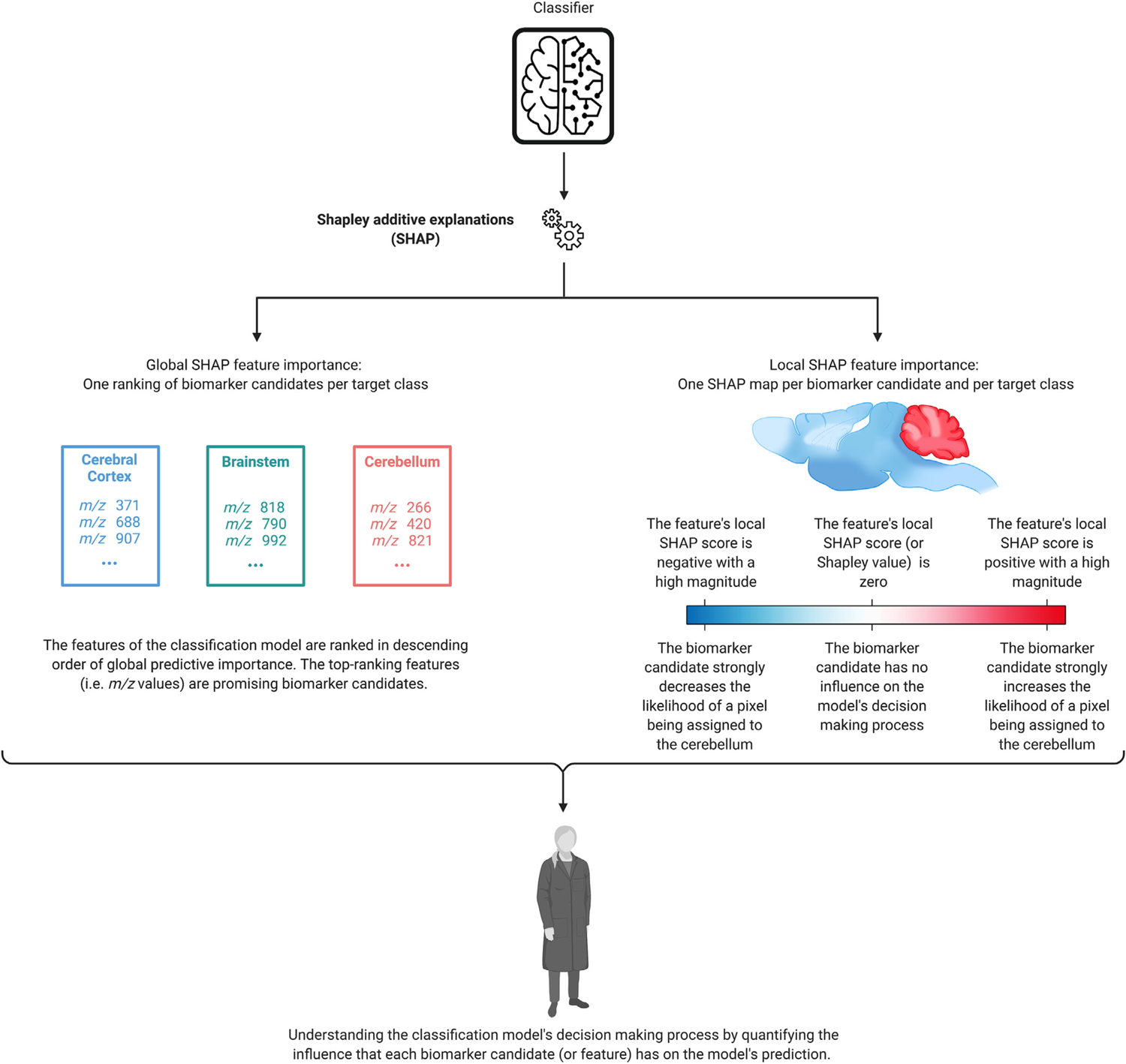
Diagram of the classifier interpretation process. SHAP is used to measure the local and global predictive importance of the features that the classifier from Fig. 1 uses to assign the pixels making up the sample surface (and their corresponding mass spectra) to one of four different anatomical classes (cerebral cortex, cerebellum, brainstem, or other). The global SHAP scores provide an experiment-wide measure of each biomarker candidate’s relevance, whereas the local SHAP scores measure the direction and magnitude of each biomarker candidate’s influence on the model output for one single pixel. SHAP maps deliver spatially localized explanations of the classifier’s decision-making process. Icons from Refs. [21,22].

In addition to automatically establishing an experiment-wide biomarker candidate shortlist by means of global SHAP score ranking, we furthermore introduce a novel spatially-aware representation of local SHAP-based explanations, called a SHAP map. The SHAP map of a molecular feature is obtained by plotting that feature’s local SHAP importance scores, or Shapley values, across all pixels. SHAP maps facilitate a spatially localized understanding of a classifier’s decision-making process. In the context of biomarker candidate discovery, SHAP maps provide one with a nuanced and location-specific (e.g. cell type specific, tissue region specific) view into a molecular species’ biomarker potential. Fig. 2 illustrates how SHAP maps can complement feature rankings for the purpose of biomarker candidate discovery. Unlike global SHAP importance scores, local SHAP importance scores avoid conflating the magnitude of the feature’s effect with the prevalence of its effect across the sample surface area.

The SHAP map of a feature answers the following two questions:

- Where does the feature increase or decrease the classifier’s output?

The feature increases the probability of the classifier assigning a pixel to the class of interest (i.e. the positive class) where its local SHAP scores are positive (red pixels). The feature decreases the probability of the classifier assigning a pixel to the positive class where its local SHAP scores are negative (blue pixels). In our application, studying the sign of a feature’s Shapley values together with the feature’s spatial distribution (e.g. the feature’s ion image) enables the user to determine whether it is the presence or the absence of a feature that is indicative of the biological state or disease condition of interest. If the regions where the feature’s measured intensity is high coincide with the regions where the feature’s Shapley values are positive, the feature’s presence is indicative of the class of interest. The relationship between the feature’s abundance and the class prediction is correlative. Conversely, if the regions where the feature’s measured intensity is low coincide with the regions where the feature’s Shapley values are positive, the feature’s absence is indicative of the class of interest. The relationship between the feature’s abundance and the class prediction is anticorrelative.

- Where does the feature strongly or weakly influence the classifier’s output?

The feature has a relatively large influence on the classifier where its Shapley values have a high magnitude (pixels with high saturation). Conversely, the feature has a relatively small influence on the model where its Shapley values have a low magnitude (pixels with low saturation). Studying the magnitude of a feature’s Shapley values provides insight into how large or small the feature’s local influence on a classifier is. In our application, we consider a feature (i.e. *m/z* value) to be relevant to recognizing a given class in the regions of the sample where its Shapley values have a high magnitude.

## 3. Results & discussion

Our biomarker candidate discovery workflow is demonstrated on two IMS datasets that were acquired by matrix assisted laser desorption/ionization (MALDI) quadrupole time-of-flight (Q-TOF) IMS using a prototype MALDI timsToF Pro (Bruker Daltonics, Germany) in positive ion mode [67]. Please refer to the supplementary material for information regarding the materials, sample preparation, experiments, histology, and IMS data preprocessing. Since the following five case studies do not involve the study of diseased tissue, the ranked features are not indicative of any pathological processes but rather of anatomical structures. Therefore, the term “molecular marker” is preferred over the term “biomarker” in Section 3. It should be noted that, methodologically speaking, there is no difference: in both cases our workflow looks for differentiating markers (i.e. *m/z* values) that correspond to user-provided classes of interest.

**Fig. 3.**
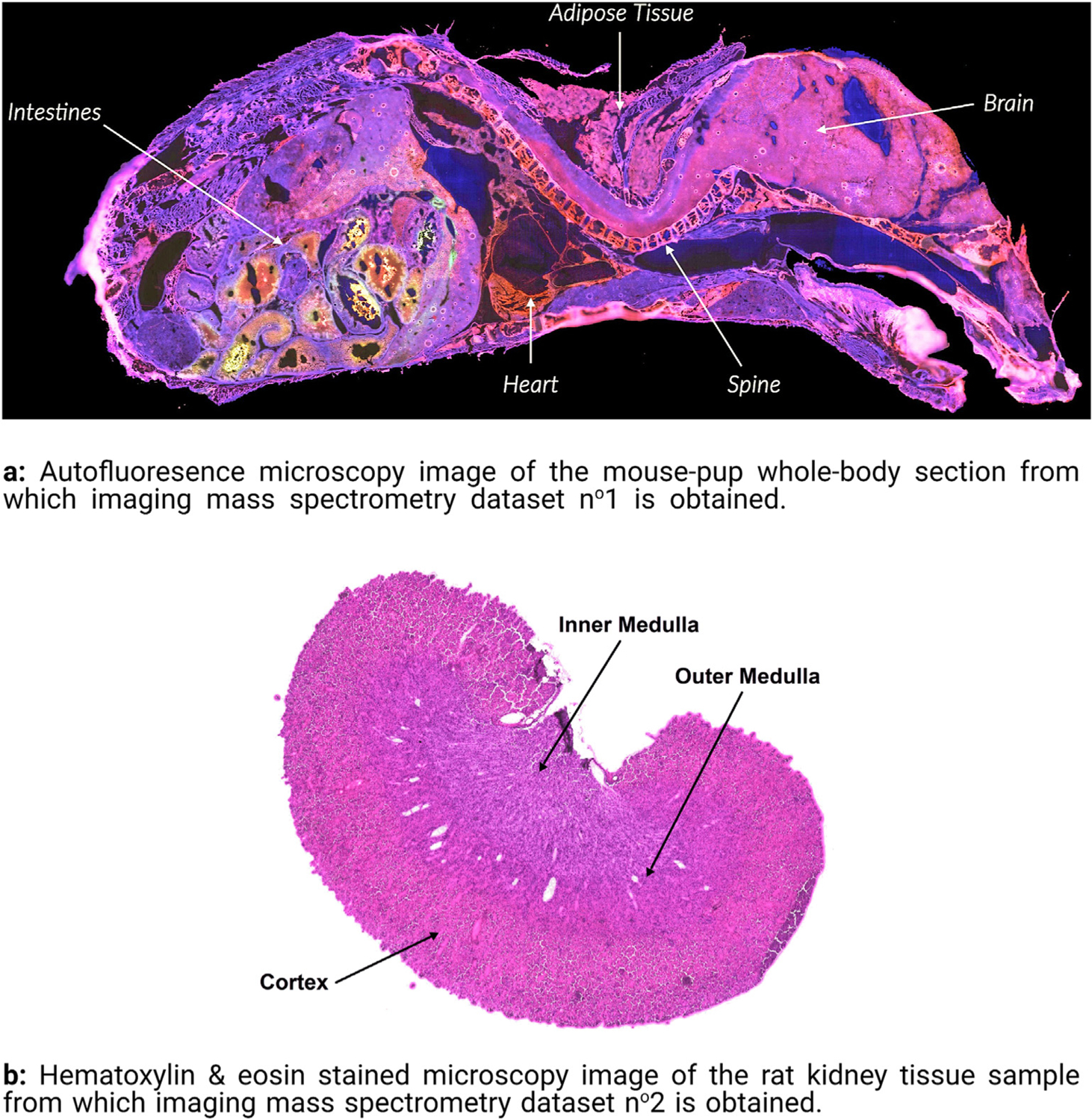
Microscopy images of the tissue sections imaged in IMS datasets n^o^1 and n^o^2.

• Dataset n^o^1 was acquired from the sagittal whole-body section of a mouse-pup. The autofluorescence microscopy image of the tissue section is presented in Fig. 3a and was used to guide annotation of the regions of interest [68]. The sample was cryosectioned at 20 mm thickness and a 1,5-diaminonaphthalene matrix was applied by sublimation. The mean mass spectrum of the dataset was retrieved and peak-picked to produce a feature list of 879 distinct ion species. The *m/z* acquisition range is 300–1200 and the pixel size is 50 μm × 50 μm. The dataset consists of a total of 164,808 pixels. Our workflow is therefore applied to a dataset of 164,808 observations and 879 features. The challenge of molecular marker discovery in the two case studies tied to this dataset therefore amounts to automatically determining which molecular species, among the 879 measured *m/z* values, are most relevant to recognizing two anatomical regions: the mouse-pup’s brain and its liver.

• Dataset n^o^2 was acquired from the sagittal section of a rat kidney. The hematoxylin & eosin stained microscopy image of the tissue section is presented in Fig. 3b. The sample was cryosectioned at 12 mm thickness and a 1,5-diaminonaphthalene matrix was applied by sublimation. The mean mass spectrum of the dataset was retrieved and peak-picked to produce a feature list of 1428 distinct ion species. The *m/z* acquisition range is 300–2000 and the pixel size is 15 μm × 15 μm. Our workflow is applied to a data table of 591,534 observations and 1428 features. The challenge of molecular marker discovery amounts to automatically determining which molecular species, among the 1428 measured *m/z* values, are most relevant for recognizing three different regions of the kidney: the cortex, the inner and outer medulla.

Five anatomical regions were delineated within the two tissue samples on the basis of the microscopy images in Fig. 3. Each tissue region was given a class label: a brain and a liver region in dataset n^o^1 and a cortex, inner medulla, and outer medulla region in dataset n^o^2. Our aim is to discover molecular markers for each of these user-provided class labels. The molecular marker discovery is treated separately for each class, using the one-versus-all procedure, yielding five binary classification problems whose target (i.e. positively labeled) classes are the following: the mouse-pup’s brain and liver in dataset n^o^1; the rat kidney’s inner medulla, outer medulla, and cortex in dataset n^o^2. Note that, although user-defined masks are employed to label the data in our case studies, our approach would work equally well if provided with automatically generated class annotations (e.g. tissue segmentation masks generated by clustering algorithms, masks provided by algorithms performing automated recognition in microscopy images). Two case studies (i.e. discovering molecular markers for the mouse-pup’s brain in dataset n^o^1, discovering molecular markers for the rat kidney’s inner medulla in dataset n^o^2) are covered in Section 3, whereas the remaining three case studies (i.e. discovering molecular markers for the mouse-pup’s liver in dataset n^o^1, discovering molecular markers for the rat kidney’s outer medulla and cortex in dataset n^o^2) are provided in the supplementary material.

As discussed in subsection 2.1, XGBoost models are used to classify the pixels on the basis of their mass spectra. These five classification problems are imbalanced because their corresponding datasets have unequal class cardinality (i.e. the negatively labeled pixels outnumber the positively labeled pixels). We avoid using accuracy (i.e. the proportion of predictions that are correct) to measure the classifier’s predictive performance since accuracy tells us little about whether false negatives or false positives are more common [69]. Instead, we choose to measure our classifier’s predictive performance using balanced accuracy, precision, and recall. Recall (also called sensitivity or the true positive rate) is the proportion of positive observations that are correctly identified. Precision is the proportion of all positive predictions that are correct. Specificity (also called the true negative rate) is the proportion of negative observations that are correctly identified. Balanced accuracy is the arithmetic mean of sensitivity and specificity [69]. As discussed in subsections 2.2 and 2.3, the TreeSHAP implementation of the SHAP interpretability method (with observational Shapley values) is used to rank the features (i.e. *m/z* values) in descending order of global predictive importance. The top-ranking features are highly discriminative with regards to a labeled tissue class and are therefore considered to be promising molecular markers for that class of interest. In addition to automatically establishing a shortlist of molecular species that are statistically related to user-provided tissue class labels, our workflow delivers spatially localized insight into the relationship (e.g. correlative, anticorrelative) between each measured ion species and the class of interest by means of a novel visualization approach called SHAP maps.

### 3.1. Dataset n^o^1: Recognition of the brain and liver of a mouse-pup

**Fig. 4.**
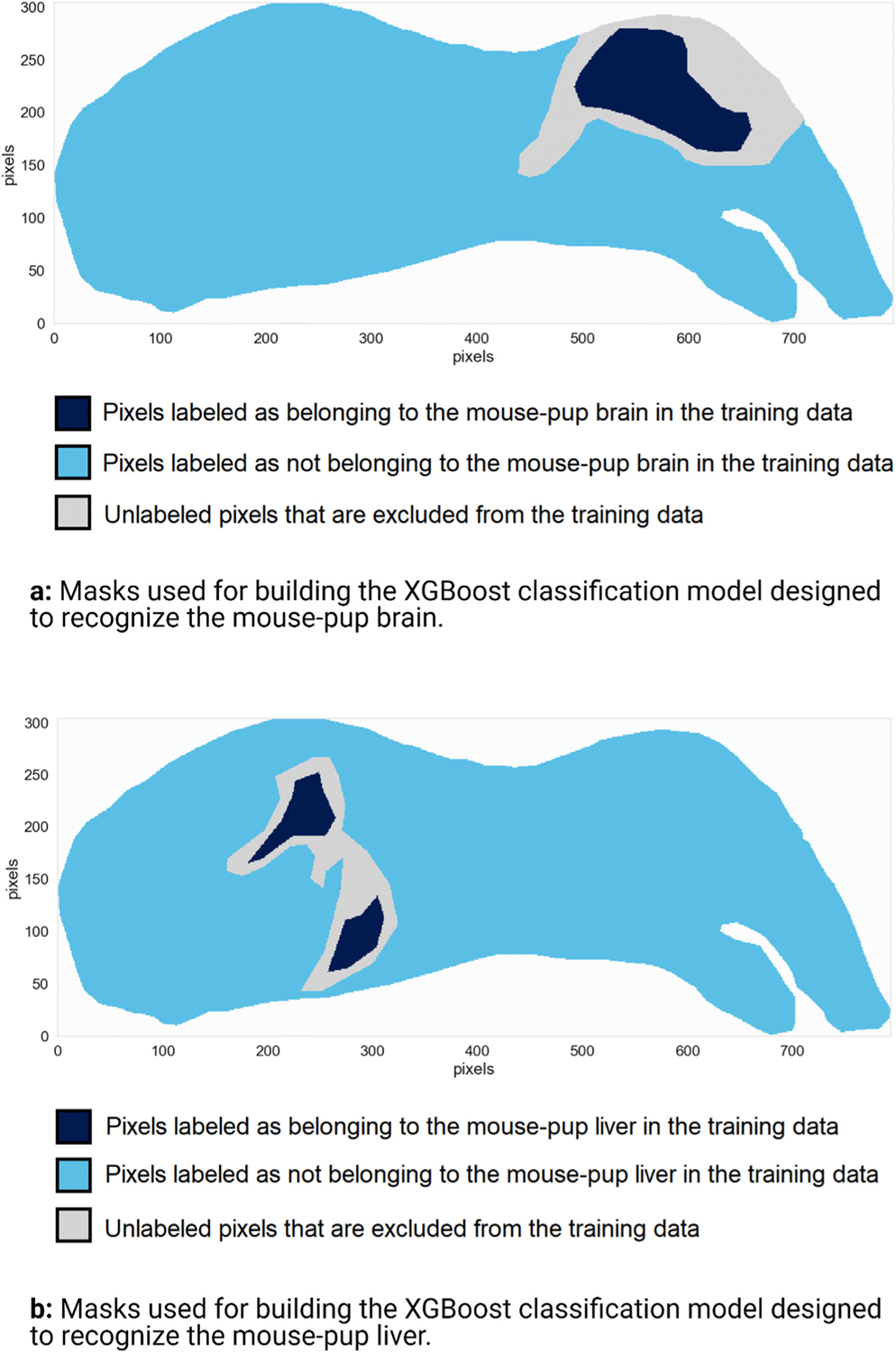
Class-defining masks used as inputs for training the two XGBoost classifiers designed to recognize the mouse-pup brain and liver. For each task, regions of the tissue sample were manually annotated as belonging to one of three categories: dark blue pixels are labeled as belonging to the target organ and make up the positive class, light blue pixels are labeled as not belonging to the target organ and make up the negative class, and gray pixels are close to borders between the target organ and other anatomical structures, making it difficult to annotate them definitively. The latter are therefore excluded from the training data to avoid feeding the supervised machine learning algorithm unreliable annotations during classifier training.

Classification-oriented supervised ML algorithms require labeled training data (in our case, labeled pixels) to build a classifier. In the two mouse-pup case studies, anatomical class labels are obtained as user-provided spatial delineations of the mouse-pup’s brain and liver in the tissue sample. Exploratory analysis of the IMS data was performed using non-negative matrix factorization to aid in that delineation task [16,70,71]. Please refer to the supplementary material to see how the low-dimensional latent patterns extracted by non-negative matrix factorization from dataset n^o^1 facilitated the manual localization and annotation of the target organs. The target organs (or tissue regions, cell types, or cells) that are provided as masks to the supervised ML algorithm are also the organs (or tissue regions, cell types, or cells) for which we want to discover molecular markers. Fig. 4 shows a spatial representation of the masks used to build the XGBoost classifiers for the mouse-pup cases. Pixels are either annotated as belonging to the target organ (i.e. positive class) or not belonging to the target organ (i.e. negative class). Some pixels (e.g. at the borders of target organs) were difficult to annotate definitively and were excluded from the training set to avoid providing the supervised ML algorithm erroneous or unreliable training examples. Furthermore, the negative class was downsampled to avoid the one-versus-all classification of the brain and liver being severely imbalanced. After downsampling, approximately 25% of the pixels used to build the classifier belong to the positive class, and approximately 75% belong to the negative class. Please refer to the supplementary material for the mouse-pup liver case study.

#### 3.1.1. Molecular marker discovery for the mouse-pup brain

**Fig. 5.**
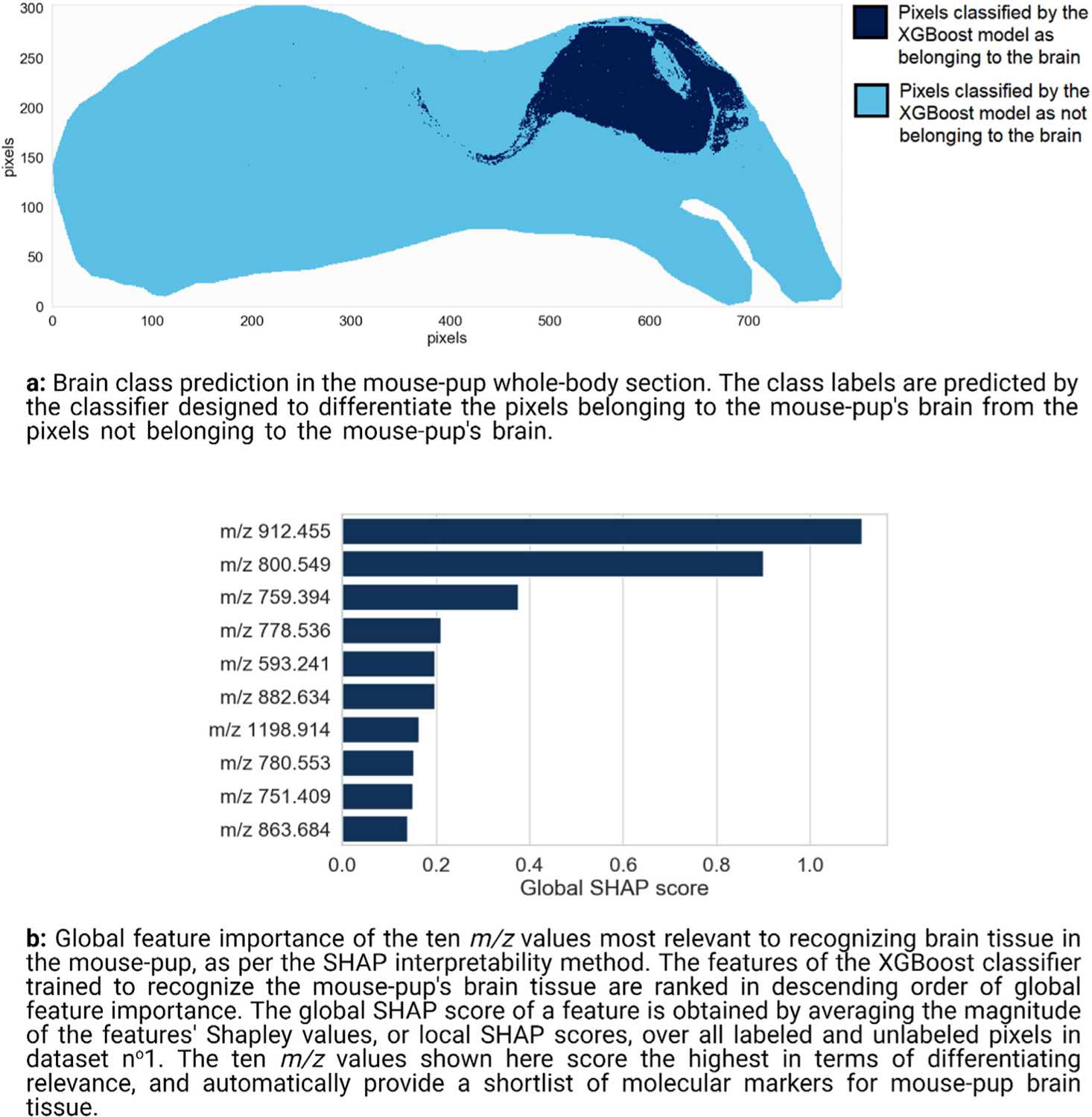
Mouse-pup brain recognition and global feature ranking.

Our brain molecular marker discovery workflow starts with building an XGBoost classifier from IMS dataset n^o^1 and the user-provided brain mask shown in Fig. 4a. Fig. 5a is the classification result obtained by supplying the classifier with all IMS measurements (both labeled and unlabeled pixels), and having it automatically recognize brain tissue pixels on the basis of their mass spectra. Fig. 5a shows which mouse-pup tissue regions are predicted to belong to the brain according to the XGBoost classifier. As is apparent from Fig. 5a, the mouse-pup’s brain (as well as parts of its spinal cord) is successfully differentiated from the other organs. Since an explanation can only be as good as its underlying model, it is necessary to verify the generalization performance of the XGBoost classifier designed to recognize pixels belonging to the mouse-pup brain. The classifier achieves a balanced accuracy of 0.9925, a precision of 0.9967, and a recall of 0.9974 on the testing dataset, which is a labeled selection of pixel measurements that is distinct from the training dataset.

**Fig. 6.**
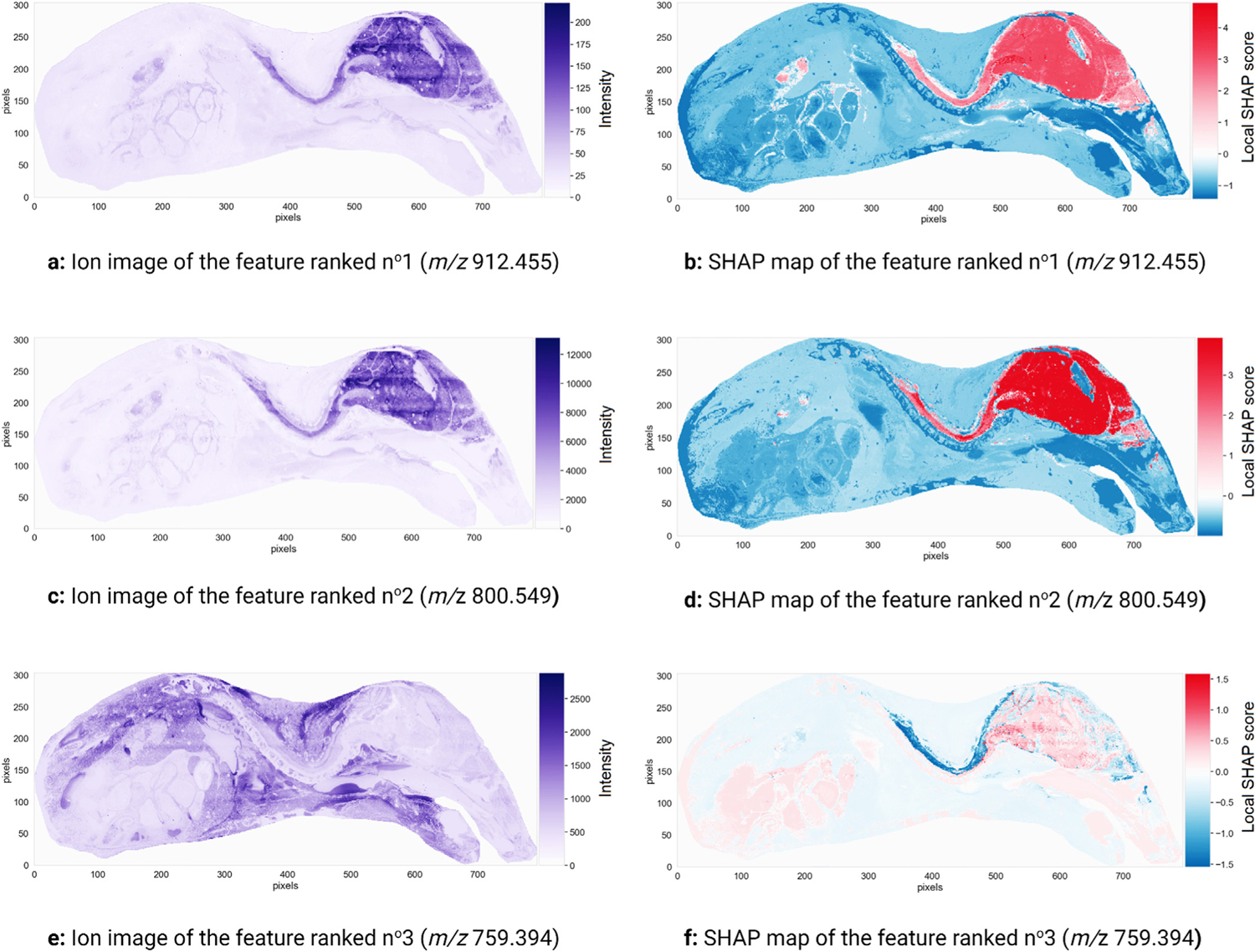
Three promising molecular markers for the mouse-pup’s brain. The ion images (left) and SHAP maps (right) of three features (i.e. *m/z* values) with the most influence on the decision-making process of the classifier trained to recognize the mouse-pup’s brain are shown. The ion images plot the spatial distribution and measured intensity of each feature across the sample, and are not specifically tied to the task of recognizing the brain. The SHAP maps plot the spatial distribution of Shapley values, or local SHAP predictive importance scores, of each feature across the sample, and provide information on where and how the feature is relevant to the task of recognizing brain.

Fig. 5b shows the top ten molecular markers of the global ranking of 879 features (i.e. *m/z* values) obtained by TreeSHAP. The features are ranked in descending order of global SHAP score, and thus in descending order of relevance to brain tissue recognition, yielding a shortlist of molecular markers for mouse-pup brain tissue. Fig. 5b provides insight into a feature’s global (i.e. tissue-wide) relevance to the recognition task of Fig. 5a. The ten top-ranking features of Fig. 5b are annotated further in Table S1 of the supplementary material, including tentative identifications. The spatially localized nature of IMS measurements together with the SHAP map representation developed above allows us to obtain tissue location specific insights into an *m/z* value’s relevance. Fig. 6 shows the ion images and SHAP maps of the three top-ranking features of Fig. 5b. The left column of Fig. 6 displays the spatial distribution and relative abundance of the three top-ranking molecular features for recognizing the mouse brain. Fig. 6a, 6c, and 6e are ion images of the features ranked n^o^1, n^o^2, and n^o^3 respectively, and they are displayed using a pseudo-color scale whose brightness is indicative of the signal intensity measured at a given pixel. These ion images provide a classical view on molecular distribution by reporting the ion intensity signal corresponding to the molecular species at hand. However, ion images do not provide any information about how that ion intensity relates to the recognition of brain tissue. The right column of Fig. 6 provides information on the signs and magnitudes of the local SHAP scores across the sample for each top-ranking feature. Fig. 6b, 6d, and 6f are the SHAP maps of the features ranked n^o^1, n^o^2, and n^o^3 respectively. These SHAP maps provide information on where and how a given ion intensity signal relates to the task of brain tissue recognition.

Fig. 6a is the ion image of the feature ranked n^o^1, whose *m/z* value is 912.455 and who has been tentatively identified as [SHexCer(38:6; O5)+K]+. The measured intensity of this feature is high in the brain and spinal cord. According to Fig. 6b, feature n^o^1 increases the log-odds (raw) output of the XGBoost classifier in the brain region: the Shapley values in the brain and spinal cord are positive, and negative elsewhere. The presence of feature n^o^1 increases the log-odds (and probability) of the classifier predicting that a given pixel belongs to the brain. The ion image and SHAP map of the feature ranked n^o^2 (*m/z* 800.549) are very similar to those of the feature in first position. Both top-ranking features are positively correlated with the classifier assigning a pixel to the brain. Measuring high intensity signals for features n^o^1 and n^o^2 in a given pixel increases the log-odds (and probability) of the classifier assigning that pixel to the brain. The high predictive performance of the XGBoost classifier suggests that it is probably a good approximation of the data generating mechanism (i.e. biochemical processes taking place in the tissue). It can therefore be assumed and inferred that measuring a high intensity signal for features n^o^1 and n^o^2 in a given pixel also increases the probability of that pixel actually belonging to the brain. In other words, the presence of these features (*m/z* values) is characteristic of the mouse-pup’s brain and spinal cord and differentiates the brain and spinal cord from other regions in the tissue.

Fig. 6e indicates that the feature ranked n^o^3, whose *m/z* value is 759.394, has a low intensity both in the brain and the spinal cord. Its measured intensity in the spinal cord is slightly higher than its intensity in the brain. Fig. 6f shows that the Shapley values of that feature are negative in the spinal cord (with a magnitude between −1.0 and −1.5). The area highlighted (negatively, hence in dark blue) in Fig. 6f, namely the spinal cord, is where feature n^o^3 plays a role in helping to obtain a biomolecular signature unique to the brain. The way in which this feature helps the classifier correctly identify the brain pixels can be read from the sign of its Shapley values, or local SHAP scores. The local SHAP values in the spinal cord are negative, meaning that whatever the signal is that is measured for this feature in the spinal cord, it lowers the log-odds (and probability) of assigning a pixel to the brain. Studying the ion image of feature n^o^3 furthermore reveals that the ion intensity for *m/z* 759.394 is low in the spinal cord, but still higher than in the brain. This means that a relative increase in signal intensity of *m/z* 759.394 strongly decreases the log-odds (and probability) of predicting a pixel belonging to the brain. Unlike the features ranked n^o^1 and n^o^2 that are good molecular markers for both the brain and spinal cord, the feature ranked n^o^3 enables the XGBoost classifier to tell the brain apart from the spinal cord. We would not be able to differentiate the mouse’s brain from its spinal cord if we were to use only the two top-ranking features (*m/z* 912.455 and *m/z* 800.549). This example illustrates the subtle understanding of molecular marker spatial specificity that can be obtained from SHAP maps. If one needs a molecular marker for both the brain and spinal cord, both *m/z* 912.455 and *m/z* 800.549 are good candidates. If one requires the ability to tell brain tissue apart from spinal cord tissue, a more elaborate panel of molecular markers is proposed: if *m/z* 912.455 and *m/z* 800.549 are present in high abundance in a tissue area, and if *m/z* 759.394 is present in very low abundance, the probability of those pixels describing brain tissue (exclusively) is very high.

### 3.2. Dataset n^o^2: Recognition of renal inner medulla, outer medulla, and cortex

**Fig. 7.**
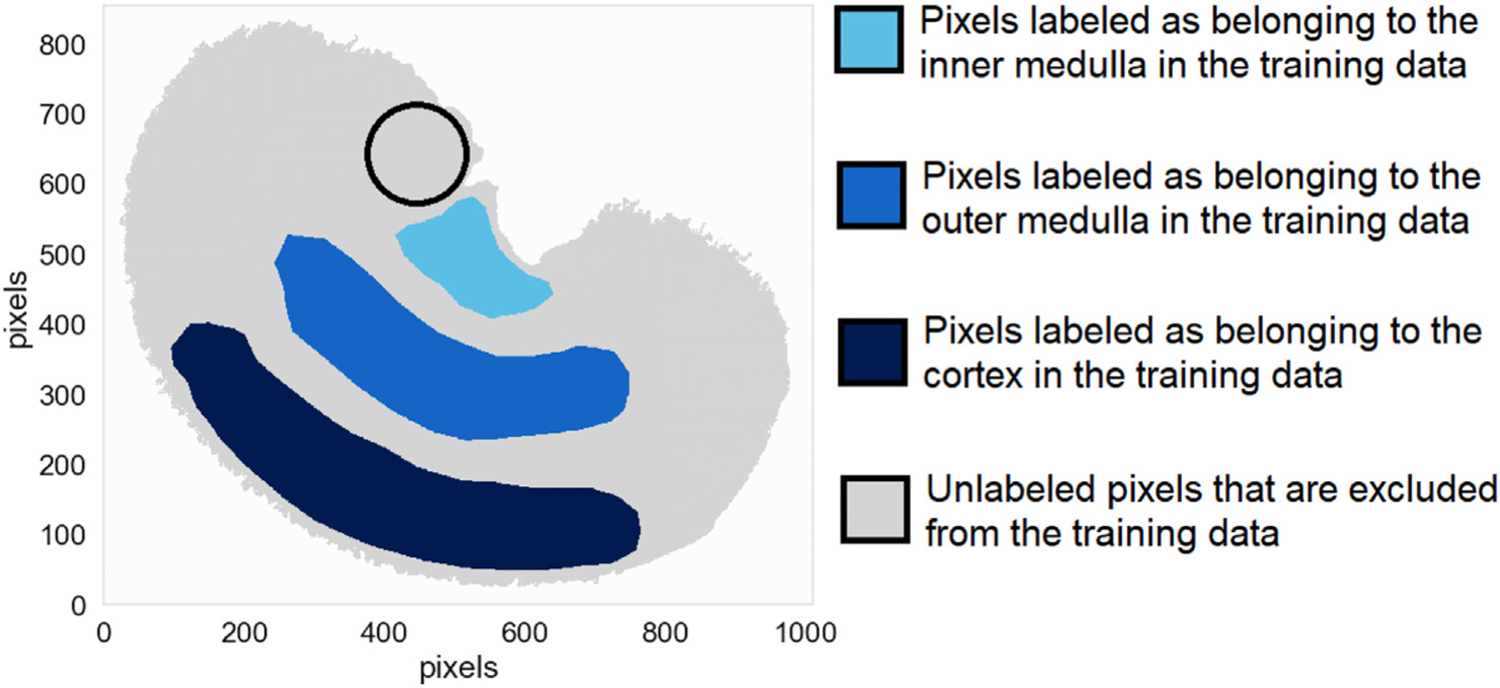
Masks used as inputs for training the three XGBoost classifiers directed at recognizing the kidney’s inner medulla, outer medulla, and cortex. Different regions of the tissue sample were manually annotated as belonging to one of four categories: light blue pixels belong to the inner medulla, medium blue pixels belong to the outer medulla, dark blue pixels belong to the cortex, and gray pixels are close to borders between these anatomical structures, making it difficult to annotate them definitively. The latter are excluded from the training data to avoid feeding the supervised machine learning algorithm unreliable annotations during classifier training. The black circle outlines a region of the renal cortex that was affected by a sample preparation artefact.

Annotating the three different functional tissue regions of the rat kidney - namely the inner medulla, outer medulla, and cortex - is required to generate the class labels needed to train the three corresponding XGBoost classifiers. Similar to the previous case studies (subsection 3.1), exploratory analysis by means of non-negative matrix factorization was used to aid in delineating masks. Fig. 7 shows the pixels annotated as belonging to one of the three target regions. The pixels that were difficult to annotate manually were excluded from the training and testing datasets. Similar to the previous case studies (subsection 3.1), downsampling of the negative class was performed. The inner medulla, outer medulla, and cortex are differentiated from the other two regions using one-versus-all classification where the target region is the positive class, and the two other regions make up the negative class. Please refer to the supplementary material for the outer medulla and cortex case studies.

#### 3.2.1. Molecular marker discovery for the renal inner medulla

**Fig. 8.**
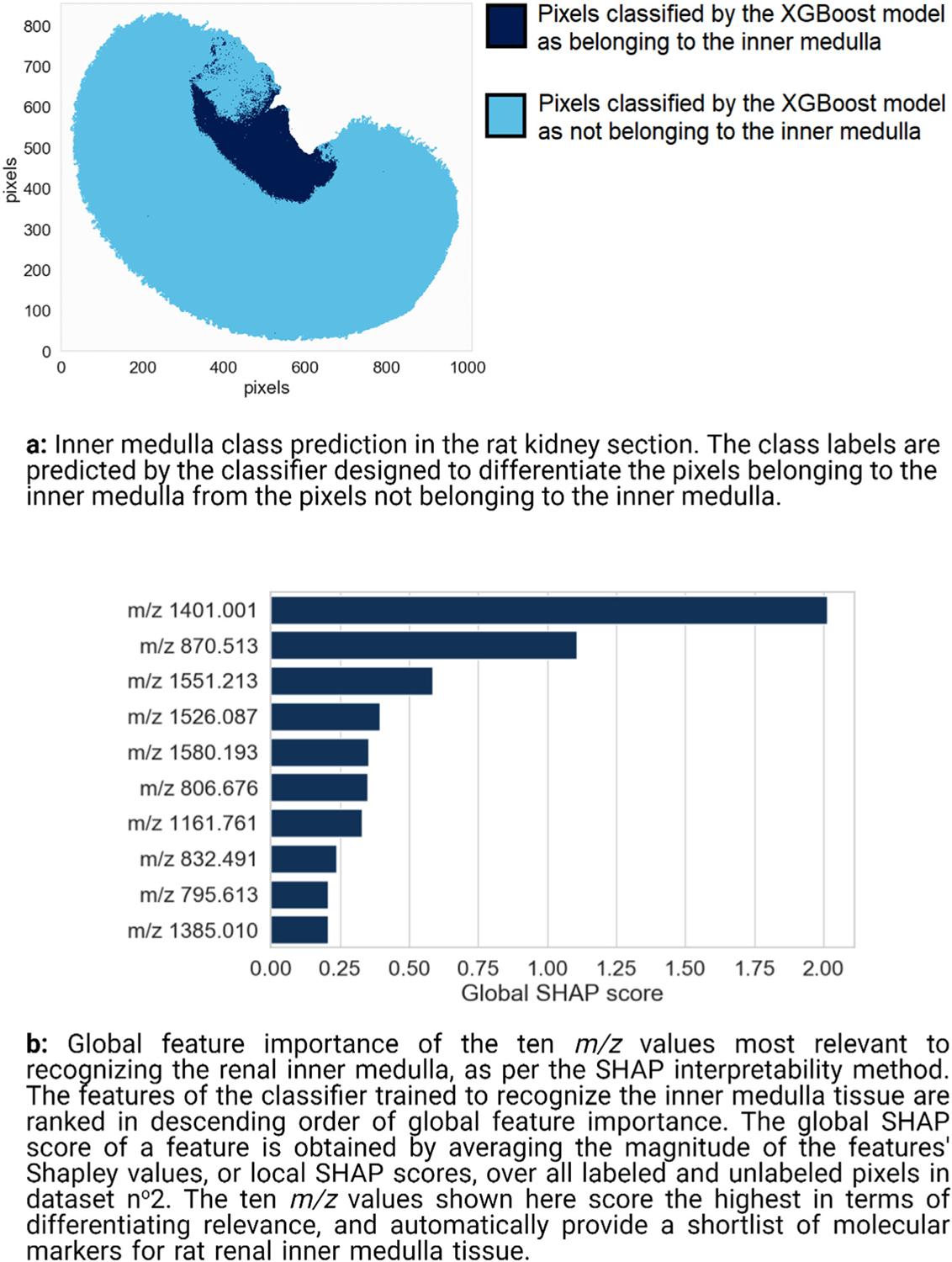
Renal inner medulla recognition and global feature ranking.

Fig. 8a presents the class prediction result for the renal inner medulla classifier, showing that the inner medulla is successfully differentiated from the outer medulla and cortex. Regarding generalization performance, the XGBoost classifier trained to recognize pixels belonging to the inner medulla achieves a balanced accuracy of 0.9992, a precision of 0.9989, and a recall of 0.9985. Note the slightly noisy region to the top-left of the medulla in Fig. 8a. The difficulties encountered by the classifier in this region, which is outlined by a black circle in Fig. 7, are probably due to a sample preparation artefact known as visceral fat delocalization [72]. Fig. 8b shows the top ten molecular markers out of the global ranking of 1428 features (i.e. *m/z* values) as obtained by TreeSHAP. The features are ranked in descending order of global SHAP score, and thus in descending order of relevance to inner medulla tissue recognition. Fig. 8b therefore provides insight into a feature’s global relevance to the recognition task of Fig. 8a. The most important feature to the XGBoost classifier used to assign pixels to the inner medulla (or not) has a *m/z* value of 1401.001 and a global SHAP score of 2.036.

**Fig. 9.**
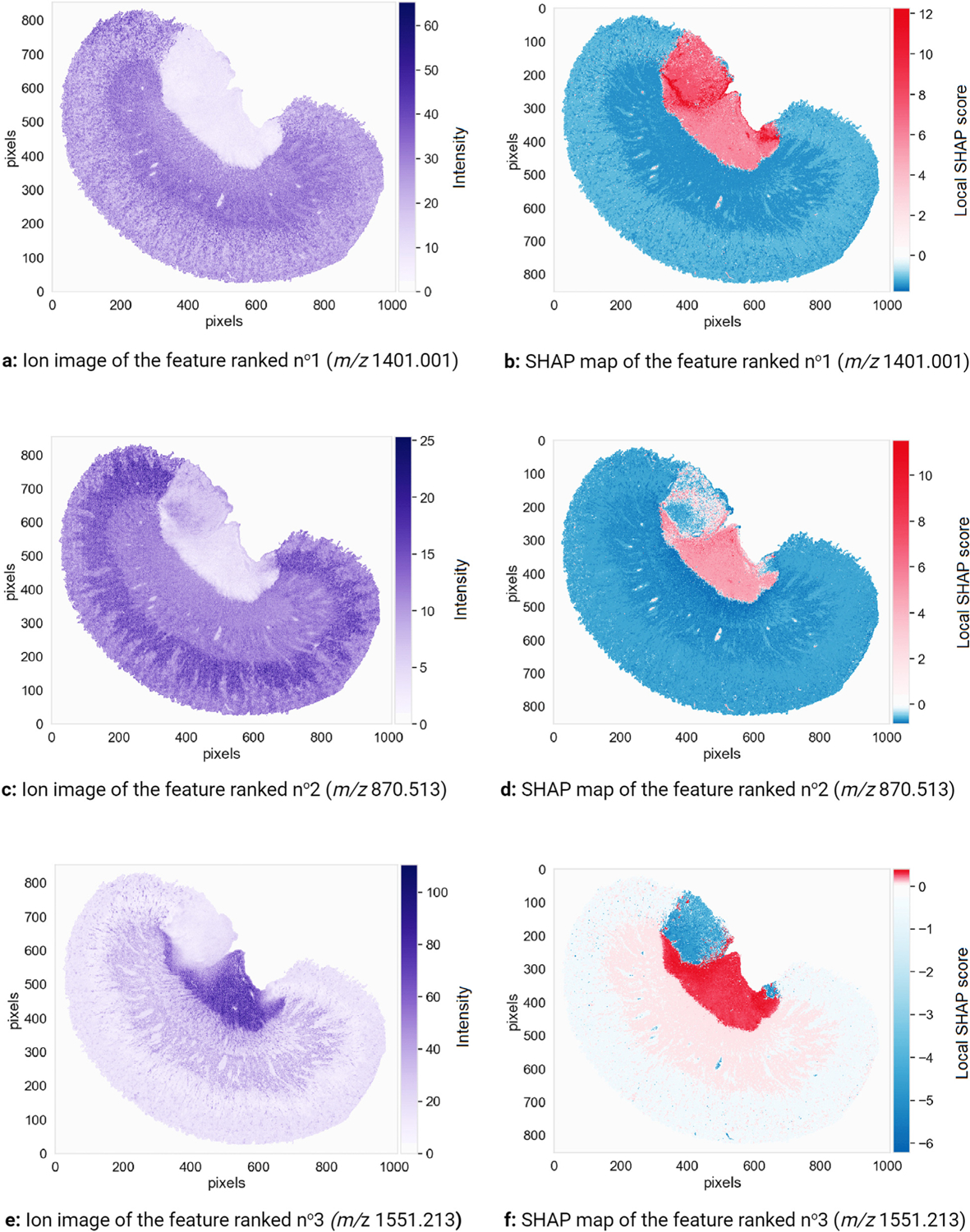
Three promising molecular markers for the renal inner medulla. The ion images (left) and SHAP maps (right) of three features (i.e. *m/z* values) with the most influence on the decision-making process of the classifier trained to recognize the rat’s renal inner medulla are shown. The ion images plot the spatial distribution and measured intensity of each feature across the sample, and are not specifically tied to the task of recognizing the inner medulla. The SHAP maps plot the spatial distribution of Shapley values, or local SHAP predictive importance scores, of each feature across the sample, and provide information on where and how the feature is relevant to the task of recognizing the inner medulla.

The left column of Fig. 9 provides information about the spatial distribution and relative abundance of the three top-ranking molecular features for recognizing the inner medulla: Fig. 9a, 9c, and 9e are the ion images of the features ranked n^o^1, n^o^2, and n^o^3 respectively. The right column of Fig. 9 provides information on the signs and magnitudes of the local SHAP scores, or Shapley values, of each top-ranking feature across the sample: Fig. 9b, 9d, and 9f are the SHAP maps of the three top-ranking features. These SHAP maps provide information on where and how a given ion intensity signal relates to the task of inner medulla tissue recognition. Combining the left and right columns of Fig. 9 provides insight into the predictive model’s decision-making process. The signal intensity measured in the inner medulla for features ranked n^o^1 and n^o^2 is low (Fig. 9a and 9c), and yet their Shapley values are high in the inner medulla (Fig. 9b and 9d). These features, *m/z* 1401.001 and *m/z* 870.513 respectively, are negatively correlated to the XGBoost classifier assigning a pixel to the inner medulla. In other words, measuring a low intensity for *m/z* 1401.001 and *m/z* 870.513 in a given pixel increases the log-odds (and probability) of the classifier assigning that pixel to the inner medulla. Given the high predictive performance of the classifier, we can assume that measuring a low intensity for *m/z* 1401.001 and *m/z* 870.513 in a given pixel also increases the probability of that pixel actually belonging to the inner medulla. Conversely, the feature ranked n^o^3 (*m/z* 1551.213) is positively correlated with the classifier predicting a pixel as belonging to the inner medulla: its intensities (Fig. 9e) and its Shapley values (Fig. 9f) are both high in the inner medulla. Measuring a high intensity for *m/z* 1551.213 in a given pixel increases the log-odds (and probability) of the classifier assigning that pixel to the inner medulla. Given the high predictive performance of the classifier, we can assume that measuring a high intensity for *m/z* 1551.213 in a given pixel increases the probability of that pixel actually belonging to the inner medulla. The absence of features ranked n^o^1 and n^o^2, and the presence of the feature ranked n^o^3 seem to be characteristic of renal inner medulla tissue.

We now focus on the tissue region to the top-left of the medulla that actually belongs to the cortex, and that was difficult for the XGBoost classifier to correctly differentiate from the inner medulla (see Fig. 8a). The SHAP map of the feature ranked n^o^1 shows (by coloring the difficult-to-classify area red) that this feature strongly increases the log-odds (and probability) of the cortex pixels to the top-left of the inner medulla being erroneously assigned to the inner medulla: the Shapley values of the feature ranked n^o^1 are positive with a high magnitude in this region of the cortex. The SHAP map of the feature ranked n^o^3 shows (by coloring the difficult-to-classify area blue) that the classifier uses this feature to correct for the labeling suggested by the feature ranked n^o^1: the Shapley values of the feature ranked n^o^3 in the region to the top-left of the inner medulla are negative with a high magnitude. This case study demonstrates an interesting level of nuance in molecular marker discovery, uniquely provided by the SHAP map representation. If only the global SHAP scores of the features are taken into account (i.e. only the global information provided in Fig. 8b, without the localized information provided in Fig. 9b, 9d, and 9f), one might be tempted to consider *m/z* 1401.001 (corresponding to the feature ranked n^o^1) as the most promising marker candidate for inner medulla tissue in this dataset. Although *m/z* 1401.001 has the most influence on the XGBoost classifier designed to recognize the inner medulla, its global SHAP score is based on a sample-wide assessment of discriminative relevance and disregards subtle spatially localized patterns. In fact, Fig. 9b shows that *m/z* 1401.001 has a positive influence on the classifier’s prediction in the inner medulla but also in a region of the cortex where visceral fat delocalization probably occurred. Unlike *m/z* 1401.001 and *m/z* 870.513 (corresponding to the features ranked n^o^1 and n^o^2 respectively), *m/z* 1551.213 (corresponding to feature ranked n^o^3) is exclusive to the inner medulla. Although the global SHAP scores of *m/z* 1401.001 and *m/z* 870.513 are higher than that of *m/z* 1551.213 (respectively 2.036 and 1.007 versus 0.548), a localized study using SHAP maps shows that *m/z* 1551.213 is the more reliable inner medulla molecular marker of the three because of its high spatial specificity. Unlike the signal of *m/z* 1551.213, the signals corresponding to *m/z* 1401.001 and *m/z* 870.513 were affected by the sample preparation artefact that took place in the renal cortex. This example also illustrates the importance of not basing one’s estimate of a molecular marker candidate’s relevance exclusively on its global SHAP predictive importance score. When visualized in the form of SHAP maps, the local SHAP scores (or Shapley values) provide useful spatially localized information as to how and where the molecular marker influences the predictive model’s output and (assuming the classifier has good predictive performance) how it ties to the underlying tissue.

## 4. Conclusion

In this work, we propose an innovative computational approach for automating the discovery of biomarker candidates in molecular imaging data. Our approach enables one to efficiently filter a multitude of molecular species down to a panel of promising biomarker candidates. Applying the automated biomarker candidate discovery workflow to imaging mass spectrometry (IMS) data is especially interesting because of the massively multiplexed nature of IMS. By enabling the untargeted concurrent mapping of hundreds to thousands of molecular species across a tissue sample, IMS enables one to cast a wide net for molecular species with biomarker potential. However, the wide range of candidates can pose difficulties since manual examination of IMS data is impractical. Automating biomarker candidate discovery in IMS using machine learning (ML) methodologies, rather than resorting to manual examination, can help re-establish the practical feasibility of IMS-based biomarker discovery, and can help maintain objectivity, scalability, and reproducibility. Our biomarker candidate discovery workflow produces a ranking of molecular species according to the discriminative relevance they hold for a given tissue structure or disease condition, such that the top-ranking molecular species are highly promising biomarker candidates that merit further study.

Our approach to biomarker candidate discovery is to identify highly discriminative molecular species whose overexpression or underexpression characterize a user-defined biological class of interest. A supervised ML algorithm, called extreme gradient boosting (XGBoost), is used to learn a classifier from labeled imaging mass spectrometry data, and a state-of-the-art ML model interpretability method, called Shapley additive explanations (SHAP), is used to measure the local and global predictive importance of the *m/z* values that the classification model uses as features. We translate the task of biomarker candidate discovery into a feature ranking problem: the features are ranked in descending order of global SHAP importance and the top-ranking features are retained for further investigation. The TreeSHAP implementation of Shapley additive explanations, with observational Shapley values, is used for quantifying the local and global predictive importance of features. In order to add nuance to our analysis, we furthermore introduce SHAP maps, a novel representation and visualization that brings a spatial dimension to our understanding of the decision-making processes of a classifier. The SHAP map of a feature is obtained by plotting that feature’s local SHAP importance scores, or Shapley values, across all pixels making up the sample surface. A feature’s local SHAP importance score is informative of the direction (e.g. positive or negative) and magnitude (e.g. large or small) of the feature’s influence on the classifier’s output for a given pixel. SHAP maps provide insight into the spatial specificity of biomarker candidates by showing how and where a feature influences the classifier’s probability of assigning a pixel, and its corresponding mass spectrum, to the class of interest.

Although our two case studies concern imaging mass spectrometry data, our biomarker candidate discovery workflow is also applicable to other forms of multiplexed imaging data such as multiplexed fluorescence microscopy (e.g. CODEX), imaging mass cytometry, near-infrared imaging, and Raman spectroscopic imaging, and therefore holds the potential to substantially advance biomarker development across a wide range of spectral imaging modalities. One area where our approach can be employed is in the discovery of clinically relevant molecular signatures for functional tissue units in the context of large-scale molecular mapping projects such as the NIH-sponsored Human BioMolecular Atlas Program [73], which aims to build a complete molecular map of the human body at single-cell resolution, and the Kidney Precision Medicine Project [74], which aims to build a comprehensive molecular, cellular, and anatomical map of the kidney. Our work on ML interpretability for multiplexed imaging may also help advance research in biomedical imaging, for example in the field of data-driven multi-modal image fusion [75], where a cross-modal regression model ties the observations in one imaging modality to the observations in another modality. Obtaining spatially-localized insight into how cross-modal connections are made holds potential for advancing all fusion applications, including prediction to a higher spatial resolution, out-of-sample predictions, as well as cross-modal denoising and relationship discovery [76].

## Supplementary Material

Supplement

## HIGHLIGHTS

- Biomarker discovery in imaging mass spectrometry data can be considered a feature ranking problem.
- Interpretability methods enable automated estimation of biomarker candidate potential.
- Our biomarker candidate discovery workflow uses Shapley additive explanations (SHAP).
- SHAP measures the direction and magnitude of a feature’s influence on a classifier.
- SHAP maps provide insight into the spatial specificity of biomarker candidates.

## Footnotes

1 There is no formal definition of supervised machine learning model interpretability that is agreed-upon within the computer science and data science communities [23–25]. The definition we propose, namely explaining a model’s decision-making process by quantifying the influence of its input features on its output, is specific to the analysis of imaging mass spectrometry data using supervised machine learning methods other than deep learning. We use the terms interpretability and explainability interchangeably.

2 Overfitting is a common issue that occurs when a regression or classification model adapts too closely to the training data and memorizes not only the relationship between inputs and outputs but also the noise. The structure of an overfit model is very sensitive to changes in its training data. Such a model usually performs poorly on new data. The risk of overfitting tends to increase when handling high-dimensional data like imaging mass spectrometry data.

3 Gini importance, which is the default measure of feature importance in Scikit-Learn’s implementation of random forest [59] and gain, which is the default measure of feature importance in the Scikit-Learn wrapper interface for XGBoost’s implementation of extreme gradient boosting [60], are two examples of popular yet inconsistent impurity-based approaches for estimating features’ predictive importances. The Gini importance of a feature is computed by averaging the weighted decrease in node impurity achieved by splitting a node using that feature over all decision trees making up the ensemble [61].

4 Permutation importance was originally developed by Breiman, under the name of mean decrease accuracy, as a model-specific method for measuring feature importance in random forests [63]. The idea is to randomly permute a feature across all observations to break its association with the model prediction (and the other features) and effectively cancel its predictive power [64]. Therefore, if the feature under study is strongly associated to the prediction, permuting its values should result in a large drop in predictive performance. Conversely, if the feature is weakly associated to the prediction, permuting it should have little to no impact on performance.

## Abbreviations:

IMS: imaging mass spectrometry
*m*/*z*: mass-to-charge ratio
MALDI: matrix assisted laser desorption/ionization
ML: machine learning
PI: permutation importance
Q-TOF: quadrupole time-of-flight
SHAP: Shapley additive explanations
XGBoost: extreme gradient boosting
