# Supplementary material for: Automated biomarker candidate discovery in imaging mass spectrometry data through spatially localized Shapley additive explanations
Source: Anal Chim Acta. Author manuscript; Available in PMC 2023 Apr 24. (PMC10124144; doi:10.1016/j.aca.2021.338522)
Supplement: Supplement [file NIHMS1892535-supplement-Supplement.pdf]

Supplementary material

Additional information for the paper entitled "Automated Biomarker Candidate Discovery in Imaging Mass Spectrometry Data Through Spatially Localized Shapley Additive Explanations" by Leonoor E.M. Tideman, Lukasz G. Migas, Katerina V. Djambazova, Nathan Heath Patterson, Richard M. Caprioli, Jeffrey M. Spraggins, and Raf Van de Plas.

Corresponding author email: raf.vandeplas@tudelft.nl

Contents:

1. Experimental protocol
2. Data preprocessing
3. Data labeling
4. Feature identification
5. Additional case studies

Experimental protocol

Materials

Acetic acid, 1,5-diaminonaphthalene (DAN), ammonium formate, hematoxylin, and eiosin were purchased from Sigma-Aldrich Chemical Co. (St. Louis, MO, USA). HPLC-grade ethanol was purchased from Fisher Scientific (Pittsburgh, PA, USA).

Sample Preparation

A one-week old C57BL/6 control mouse pup was snap frozen at -80°C, shaved over dry ice, and cryosectioned at 20 μm thickness, using a CM3050 S cryostat (Leica Biosystems, Wetzlar, Germany). The tissue was thaw-mounted onto a conductive indium tin oxide coated glass slide (Delta Technologies, Loveland, CO, USA). Rat kidney tissue was purchased from PelFreeze Biologicals (Rogers, AR, USA), sectioned at 10 μm thickness and thaw-mounted onto a conductive slide. Autofluorescence microscopy images were acquired using EGFP, DAPI, and DsRed filters on a Zeiss AxioScan Z1 slide scanner (Carl Zeiss Microscopy GmbH, Oberkochen, Germany). Approximately 500 mg of DAN was sublimed at 130°C and 24 mTorr for 3.5 min onto the tissue surface for a final density of ~1.0 mg/cm^2.

MALDI TIMS-IMS

Our biomarker candidate discovery workflow is demonstrated on two imaging mass spectrometry (IMS) datasets. All experiments were carried out on a prototype timsTOF fleX mass spectrometer (Bruker Daltonik, Bremen, Germany).

- Dataset n°1: Mouse pup images were acquired in trapped ion mobility (TIMS) mode of operation with an ion transfer time of 100 μs, prepulse storage time of 8 μs, and a collision RF of 2,000 Vpp, a TIMS funnel 1 (accumulation) RF of 450 Vpp, a TIMS funnel 2 RF (analysis) of 400 Vpp, a multipole RF of 400 Vpp, and a collision cell entrance (in) voltage of 300 V. Tissue imaging data (164,808 pixels) were collected at 50 μm pixel size, using 200 shots per pixel and 48% laser power. Data were collected in positive ionization mode from *m/z* 300 to 1,200. The TIMS scan time was set to 400 ms, with a reduced mobility ($1/K_0$) range of 0.4 - 1.9 (V·s)/cm^2.

- Dataset n°2: Murine kidney images were generated in Q-TOF mode of operation. Tissue imaging data (591,534 pixels) were collected at 15 μm pixel size, using 400 shots per pixel, and 35% laser power. Data were collected in positive ionization mode from *m/z* 200 to 1,500.

FT-ICR IMS

To aid in the identification of detected lipid species of the mouse pup dataset, we used data from parallel analysis performed on a 15T solariX FT-ICR mass spectrometer (Bruker Daltonics, Bremen, Germany) coupled to an Apollo II ESI-MALDI dual ion source. The data was collected for the *m/z* range of 460-1,500 with a transient length of 4.2 s, resulting in a resolving power of 600,000 at *m/z* 760.

Histology

Following MALDI IMS, the matrix was removed from tissue using 100% ethanol and rehydrated with graded ethanol and double distilled H2O. The tissues were then stained using a hematoxylin and eosin (H&E) stain. Brightfield microscopy of stained tissues was obtained at 20× magnification using a Leica SCN400 Brightfield Slide Scanner (Leica Microsystems, Buffalo Grove, IL, USA).

Data preprocessing

- Dataset n°1: The mouse-pup data was exported into a custom binary format optimized for storage and speed of analysis of the ion mobility-IMS data. Each frame/pixel contains between 10,000 and 100,000 centroid peaks that span the acquisition range of *m/z* 300-1,200 and $1/K_0$ 0.4-1.9 (V·s)/cm^2 with 221,888 and 4,001 bins along the mass spectrometry and ion mobility dimensions, respectively. The processing pipeline requires common *m/z* and $1/K_0$ axes, hence individual centroid peaks were inserted at their correct bin positions along the mass spectrometry and ion mobility dimensions; missing values were set to zero. Following the conversion process, the mass spectra were aligned and calibrated along the *m/z* axis to reduce peak drift and permit identification. The *m/z* alignment was driven by four reference peaks that were present throughout the dataset: 703.5748, 734.5694, 760.5851, 810.6001. After calibration, a mean mass spectrum of the entire dataset was generated and peak picked. A total of 879 features were selected and extracted to generate ion mobility-rich ion images. Since our biomarker candidate discovery workflow is optimized for non-ion mobility data, the ion mobility information is removed by summation of all ion mobility bins of the ion mobility-rich images to a single vector, resulting in a standard ion image.

- Dataset n°2: The murine kidney data was exported into a custom binary format as described above. Data were acquired in the Q-TOF only mode, hence the ion mobility dimension is not present. We introduced a trivial second spectral dimension by enforcing the dataset to contain one ion mobility bin, in order to be able to lean on recent developments for efficient storage and data processing, but this process does not have any impact on the actual data. Following the conversion process, a mean mass spectrum of the entire dataset was generated, which was furthermore peak picked resulting in a total of 1,428 ion images for distinct features in the data.

Low variance noise was removed from both datasets by principal component analysis: principal component analysis is a matrix factorization technique that can be used to reduce the dimensionality of an IMS dataset while retaining most of its original variance, and hence most of its latent molecular information [16, 77]. Feature centering, which consists in subtracting the mean from each column of the IMS data matrix, is performed without feature scaling. We argue that feature scaling prior to classifier training is not necessary because large differences in high intensity peaks are biologically informative, and high intensity peaks should therefore have more influence on the classifier than low intensity peaks [16].

Data labeling

The supervised ML algorithms used to learn classifiers from IMS data require the pixels in the training set to be labeled. In our work, the pixels belonging to different tissue functional units are labeled by user-defined masks. The low-dimensional latent patterns extracted by non-negative matrix factorization from datasets n°1 and n°2 delineated several tissue functional units within the tissue sections and therefore facilitated the manual localization and annotation of the target regions. The mouse-pup liver and brain masks of Figure 4 were defined by using MATLAB's Image Processing Toolbox to draw polygonal regions of interest, guided by the latent features displayed in Figure S1. The quality of these user-defined masks was confirmed by collaborators at Vanderbilt University. The renal inner medulla, outer medulla, and cortex masks of Figure 7 were defined using the same approach. Note that our biomarker candidate discovery workflow would work equally well if provided with automatically-generated class annotations.

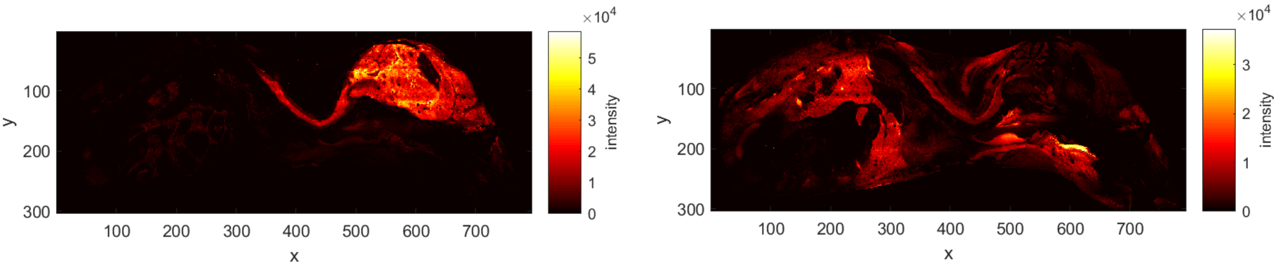

(a) Latent feature extracted using non-negative matrix factorization that highlights the mouse-pup brain.

(b) Latent feature extracted using non-negative matrix factorization that highlights the mouse-pup liver.

Figure S1. Latent features obtained from dataset n°1 by non-negative matrix factorization in order to generate the brain and liver masks displayed in Figure 4. The masks are polygons that were drawn by hand with guidance from these latent feature plots.

Feature identification

The top features discovered using our workflow were run against the LipidMaps database (lipidmaps.org) to produce tentative annotations [78, 79]. We selected $[M+H]^{+}$, $[M+Na]^{+}$, and $[M+K]^{+}$ as the adduct ions, selected the "even chains only", and specified a narrow 0.005 m/z mass tolerance window. We calculated the ppm error associated with each assignment and removed assignments with errors larger than 3 ppm. Annotations are only provided for the mouse pup dataset because many of the top selected features for the rat kidney dataset were [M+1] rather than [M] ions.

**Feature m/z**	**Feature formula**	**Feature name**
912.455	$C_{44}H_{75}O_{14}$	[SHexCer(38:6;O5)+K]+
800.5492	NA	NA
759.394	NA	NA
778.536	$C_{44}H_{77}O_{8}$	[PC(36:6)+H]+
593.241	NA	NA
882.634	$C_{48}H_{94}O_{8}$	[PC(40:1)+K]+
1198.914	NA	NA
780.553	$C_{48}H_{94}O_{8}$	[PC(36:5)+H]+
751.409	NA	NA
863.684	$C_{47}H_{96}N_{2}O_{9}$	[SM(42:1;O5)+H]+

Table S1. Tentative chemical identifications of mouse-pup brain molecular markers.

Feature m/z	Feature formula	Feature name
746.607	$C_{42}H_{85}O_7$	[PC(O-34:1)+H]+
757.622	$C_{43}H_{86}N_2O_6$	[SM(d38:2)+H]+
558.292	$C_{26}H_{50}NO_7PK$	[LPC(18:2)+K]+
718.575	NA	NA
892.661	NA	NA
732.553	$C_{40}H_{79}O_8$	[PC(32:1)+H]+
716.559	$C_{40}H_{79}O_7$	[PC(O-32:2)+H]+
733.555	$C_{40}H_{81}O_7$	[PC(O-32:1)+H]+
948.506	NA	NA
778.536	$C_{44}H_{77}O_8$	[PC(36:6)+H]+

Table S2. Tentative chemical identifications of mouse-pup liver molecular markers.

Additional case studies

Molecular marker discovery for the mouse-pup liver (dataset n°1)

Our liver molecular marker discovery workflow starts with building a classifier from IMS dataset n°1 and the user-provided liver mask shown in Figure 4b. Figure S2a shows the result of applying the classifier to all pixels of dataset n°1: the mouse-pup's liver is successfully differentiated from the other organs, demonstrating that the classifier was able to capture a liver-specific mass spectral signature in the data. Regarding generalization performance, the XGBoost classifier trained to recognize pixels belonging to the mouse-pup liver achieves a balanced accuracy of 0.9960, a precision of 0.9984, and a recall of 0.9989.

Figure S2b shows the top ten molecular markers out of the global ranking of 879 features (i.e. *m/z* values) as obtained by TreeSHAP. The features are ranked in descending order of global SHAP predictive importance score, and thus in descending order of relevance to liver tissue recognition. Figure S2b therefore provides insight into a feature's global relevance to the recognition task of Figure S2a. The feature with the highest influence on the XGBoost classifier used to assign pixels to the liver (or not) has a *m/z* value of 746.607 and a global SHAP score of 1.595. The left column of Figure S3 provides information about the spatial distribution and relative abundance of the three top-ranking molecular features for recognizing the mouse liver in this dataset. Figures S3a, S3c, and S3e are the ion images of the features ranked n°1, °2, and °3 respectively. The right column of Figure S3 provides information on the signs and magnitudes of the local SHAP scores, or Shapley values, across the sample for each top-ranking feature. Figures S3b, S3d, and S3f are the SHAP maps of the three top-ranking features. These SHAP maps provide information on where and how a given ion intensity signal relates to the task of liver tissue recognition. Studying the left and the right columns of Figure S3 enables one to understand which features are positively or negatively correlated with a pixel being assigned to the liver.

The ion image of the feature ranked n°1 (Figure S3a) indicates that it has a very low abundance in the liver region. Yet the SHAP map of that feature (Figure S3b) shows that the local SHAP scores are positive, with a high magnitude, in the liver region. The feature ranked n°1 can therefore be considered negatively correlated with a pixel being assigned to the liver. The feature ranked n°2 is also negatively correlated with the XGBoost classifier assigning a pixel to the liver. In short, measuring a low intensity for features ranked n°1 and n°2 in a given pixel increases the log-odds (and probability) of the classifier assigning that pixel to the liver. Given the high predictive performance of the classifier, we can assume that measuring a low intensity of features ranked n°1 and n°2 in a given pixel also increases the probability of that pixel actually belonging to the liver. Conversely, the feature ranked n°3 is positively correlated with a pixel being assigned to the liver: its ion image (Figure S3e) indicates that the feature ranked n°3 has a high abundance in the liver, and its SHAP map (Figure S3f) indicates that its local SHAP scores are high in the liver region. Measuring a high intensity of the feature ranked n°3 in a given pixel increases the log-odds (and probability) of the classifier assigning that pixel

to the liver. Furthermore, given the high predictive performance of the classifier, we can assume that measuring a high intensity of the feature ranked n°3 in a given pixel also increases the probability of that pixel actually belonging to the liver. The absence of features ranked n°1 and n°2, and the presence of the feature ranked n°3 are therefore characteristic of the mouse-pup liver. If one measures a low abundance of *m/z* 746.607 and *m/z* 757.622 and a high abundance of *m/z* 558.292 in a pixel, the probability of that pixel belonging to the liver is very high.

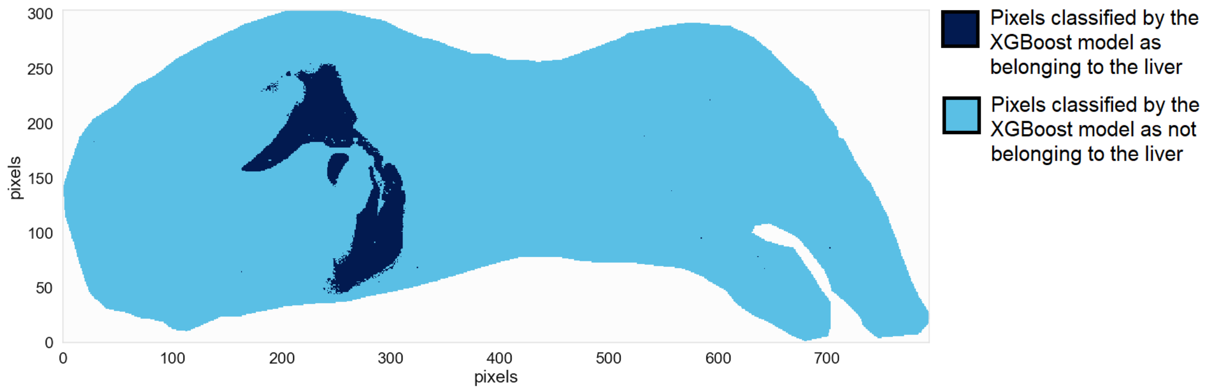

(a) Liver class prediction in the mouse-pup whole-body section. The class labels are predicted by the classifier designed to differentiate the pixels belonging to the mouse-pup's liver from the pixels not belonging to the mouse-pup's liver.

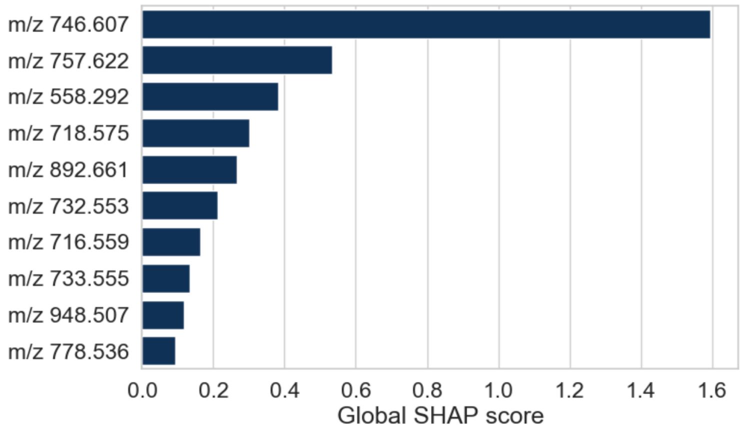

(b) Global feature importance of the ten *m/z* values most relevant to recognizing liver tissue in the mouse-pup, as per the SHAP interpretability method. The features of the classifier trained to recognize the mouse-pup's liver tissue are ranked in descending order of global feature importance. The global SHAP score of a feature is obtained by averaging the magnitude of the feature's Shapley values, or local SHAP scores, over all labeled and unlabeled pixels in dataset n°1. The ten *m/z* values shown here score the highest in terms of differentiating relevance, and automatically provide a shortlist of molecular markers for mouse-pup liver tissue.

Figure S2. Mouse-pup liver recognition and global feature ranking.

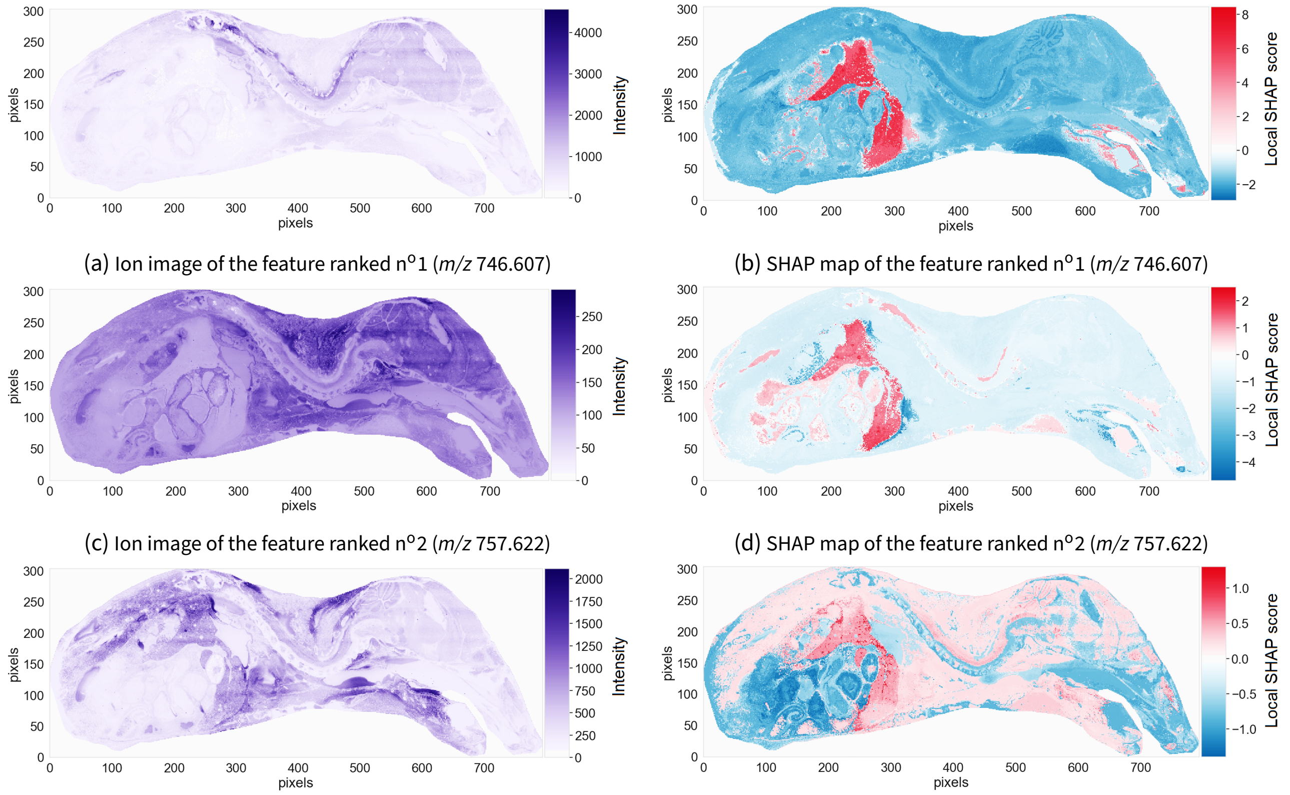

(a) Ion image of the feature ranked n°1 (*m/z* 746.607)

(b) SHAP map of the feature ranked n°1 (*m/z* 746.607)

(c) Ion image of the feature ranked n°2 (*m/z* 757.622)

(d) SHAP map of the feature ranked n°2 (*m/z* 757.622)

(e) Ion image of the feature ranked n°3 (*m/z* 558.292)

(f) SHAP map of the feature ranked n°3 (*m/z* 558.292)

Figure S3. Three promising molecular markers for the mouse-pup's liver. The ion images (left) and SHAP maps (right) of three features (i.e. *m/z* values) with the most influence on the decision-making process of the classifier trained to recognize the mouse-pup's liver are shown. The ion images plot the spatial distribution and measured intensity of each feature across the sample, and are not specifically tied to the task of recognizing the liver. The SHAP maps plot the spatial distribution of Shapley values, or local SHAP predictive importance scores, of each feature across the sample, and provide information on where and how the feature is relevant to the task of recognizing the liver.

Biomarker candidate discovery for the outer renal medulla (dataset n°2)

Figure S4a presents the classification result: the outer medulla has successfully been differentiated from the cortex and inner medulla. Regarding generalization performance, the XGBoost classifier trained to recognize pixels belonging to the outer renal medulla achieves a balanced accuracy of 0.9970, a precision of 0.9972, and a recall of 0.9958.

Figure S4b shows the top ten molecular markers out of the global ranking of 1,428 features (i.e. *m/z* values) as obtained by TreeSHAP. The features are ranked in descending order of global SHAP score, and thus in descending order of relevance to outer medulla tissue recognition. The classifier used to assign pixels to the outer medulla (or not) relies heavily on the feature ranked n°1, whose *m/z* value is 588.468 and whose global SHAP score of 2.311. The left column of Figure S5 provides information about the spatial distribution and relative abundance of the three top-ranking molecular features for recognizing the outer medulla. The right column of Figure S5 provides information on the signs and magnitudes of the local SHAP scores, or Shapley values, of each top-ranking feature across the sample. These SHAP maps provide information on where and how a given ion intensity signal relates to the task of outer medulla tissue recognition.

The feature ranked n°1 is strongly positively correlated with assigning a pixel to the outer medulla because its ion image (Figure S5a) shows a high intensity in the outer medulla and because its SHAP map (Figure S5b) shows positive Shapley values in the outer medulla and negative Shapley values in the inner medulla and cortex. Given the high predictive performance of the classifier, we can assume that measuring a high intensity of the feature ranked n°1 in a given pixel also increases the probability of that pixel actually belonging to the outer medulla. Features ranked n°2 and

n°3 (*m/z* 1444.101 and *m/z* 1543.214 respectively) are negatively correlated with the classifier assigning a pixel to the outer medulla. Given the high predictive performance of the classifier, we can assume that measuring a low intensity of the features ranked n°2 and n°3 in a given pixel also increases the probability of that pixel actually belonging to the outer medulla. The SHAP maps of features ranked n°2 and n°3 (Figures S5d and S5f respectively) show that the XGBoost classifier uses the feature ranked n°2 to differentiate the outer medulla from the inner medulla and cortex, and that it uses the feature ranked n°3 to differentiate the outer medulla and inner medulla from the cortex.

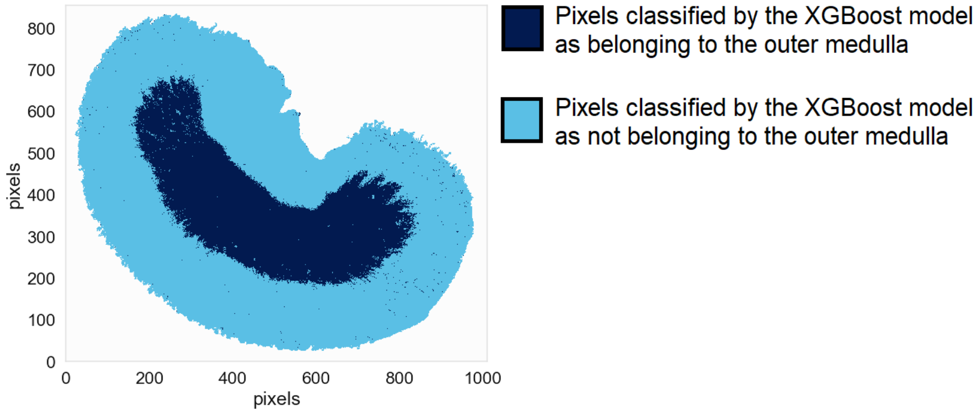

(a) Outer medulla class prediction in the rat kidney section. The class labels are predicted by the classifier designed to differentiate the pixels belonging to the outer medulla from the pixels not belonging to the outer medulla.

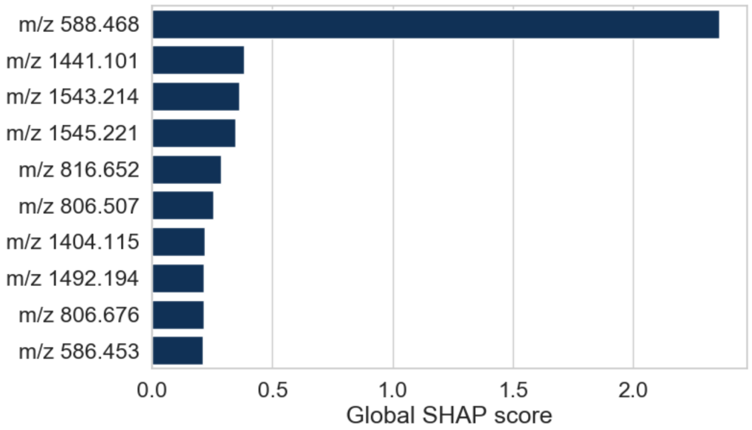

(b) Global feature importance of the ten *m/z* values most relevant to recognizing the renal outer medulla, as per the SHAP interpretability method. The features of the classifier trained to recognize the outer medulla tissue are ranked in descending order of global feature importance. The global SHAP score of a feature is obtained by averaging the magnitude of the feature's Shapley values, or local SHAP scores, over all labeled and unlabeled pixels in dataset n°2. The ten *m/z* values shown here score the highest in terms of differentiating relevance, and automatically provide a shortlist of biomarker candidates for rat renal outer medulla tissue.

Figure S4. Renal outer medulla recognition and global feature ranking.

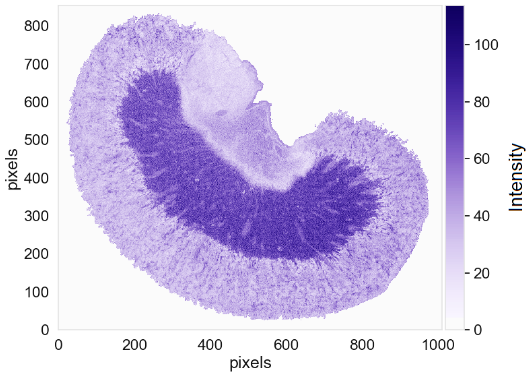

(a) Ion image of the feature ranked n°1 (*m/z* 588.468)

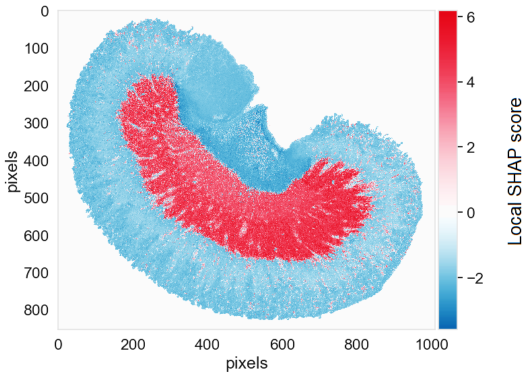

(b) SHAP map of the feature ranked n°1 (*m/z* 588.468)

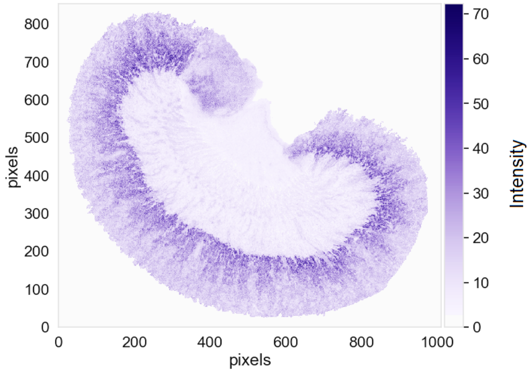

(c) Ion image of the feature ranked n°2 (*m/z* 1444.101)

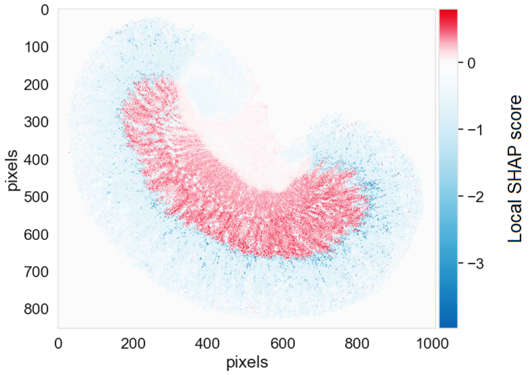

(d) SHAP map of the feature ranked n°2 (*m/z* 1444.101)

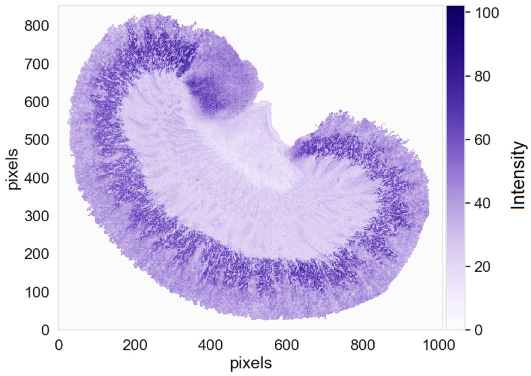

(e) Ion image of the feature ranked n°3 (*m/z* 1543.214)

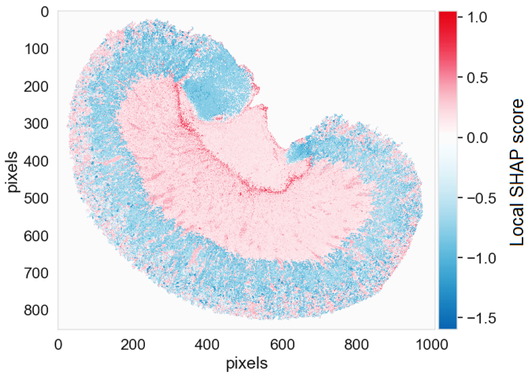

(f) SHAP map of the feature ranked n°3 (*m/z* 1543.214)

Figure S5. Three promising molecular markers for the renal outer medulla. The ion images (left) and SHAP maps (right) of three features (i.e. *m/z* values) with the most influence on the decision-making process of the classifier trained to recognize the rat's renal outer medulla are shown. The ion images plot the spatial distribution and measured intensity of each feature across the sample, and are not specifically tied to the task of recognizing the outer medulla. The SHAP maps plot the spatial distribution of Shapley values, or local SHAP predictive importance scores, of each feature across the sample, and provide information on where and how the feature is relevant to the task of recognizing outer medulla.

Biomarker candidate discovery for the renal cortex (dataset n°2)

Figure S6a presents the classification result. The cortex has successfully been differentiated from the inner and outer medulla. Regarding generalization performance, the XGBoost classifier trained to recognize pixels belonging to the renal cortex achieves a balanced accuracy of 0.9974, a precision of 0.9976, and a recall of 0.9973.

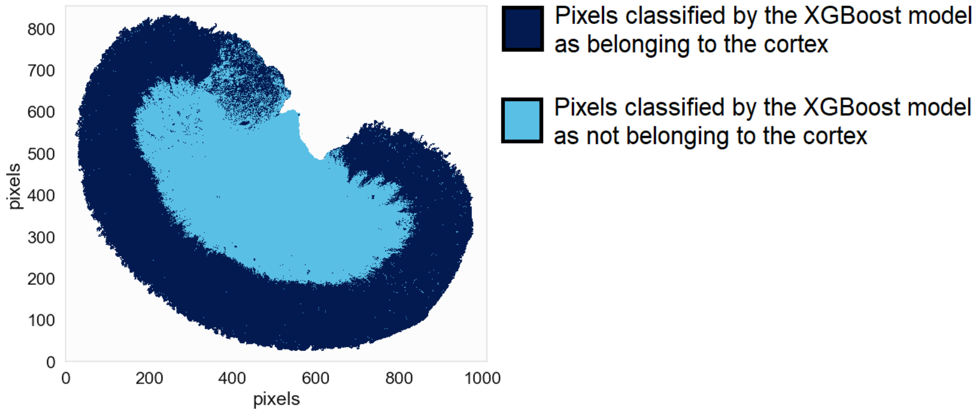

(a) Cortex class prediction in the rat kidney section. The class labels are predicted by the classifier designed to differentiate the pixels belonging to the cortex from the pixels not belonging to the cortex.

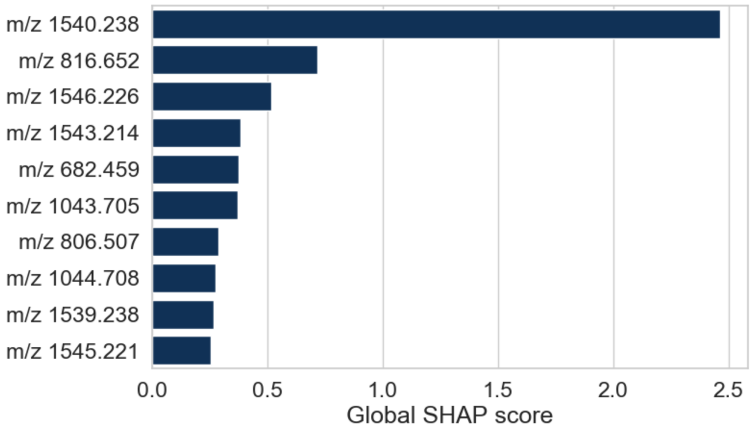

(b) Global feature importance of the ten *m/z* values most relevant to recognizing the renal cortex, as per the SHAP interpretability method. The features of the classifier trained to recognize the cortex tissue are ranked in descending order of global feature importance. The global SHAP score of a feature is obtained by averaging the magnitude of the feature's Shapley values, or local SHAP scores, over all labeled and unlabeled pixels in dataset n°2. The ten *m/z* values shown here score the highest in terms of differentiating relevance, and automatically provide a shortlist of biomarker candidates for rat renal cortex tissue.

Figure S6. Renal cortex recognition and global feature ranking.

Figure S6b shows the top ten molecular markers out of the global ranking of 1,428 features (i.e. *m/z* values) as obtained by TreeSHAP. The features are ranked in descending order of global SHAP score, and thus in descending order of relevance to cortex tissue recognition. The XGBoost model used to assign pixels to the cortex (or not) relies heavily upon one feature, whose *m/z* value is 1540.238 and whose global SHAP score is 2.507. The left column of Figure S7 presents the ion images of the three top-ranking features. The right column of Figure S7 presents the SHAP maps of the three top-ranking features. The ion image of the feature ranked n°1 (Figure S7a) shows that feature ranked n°1 has high intensity in the cortex; and the SHAP map of feature ranked n°1 (Figure S7b) shows that feature ranked n°1 has positive Shapley values in the cortex, and negative Shapley values in the inner and outer medulla. Therefore, feature ranked n°1 (i.e. *m/z* 1540.238) is positively correlated to a pixel being assigned to the cortex. Conversely, features ranked n°2 and n°3 (i.e. *m/z* 816.652 and *m/z* 1546.226 respectively) are negatively correlated with the classifier assigning a pixel to the cortex. Given the high predictive performance of the classifier, we can assume that measuring a high intensity of feature ranked n°1, and a low intensity of features ranked n°2 and n°3, increases the probability of that pixel actually belonging to the cortex.

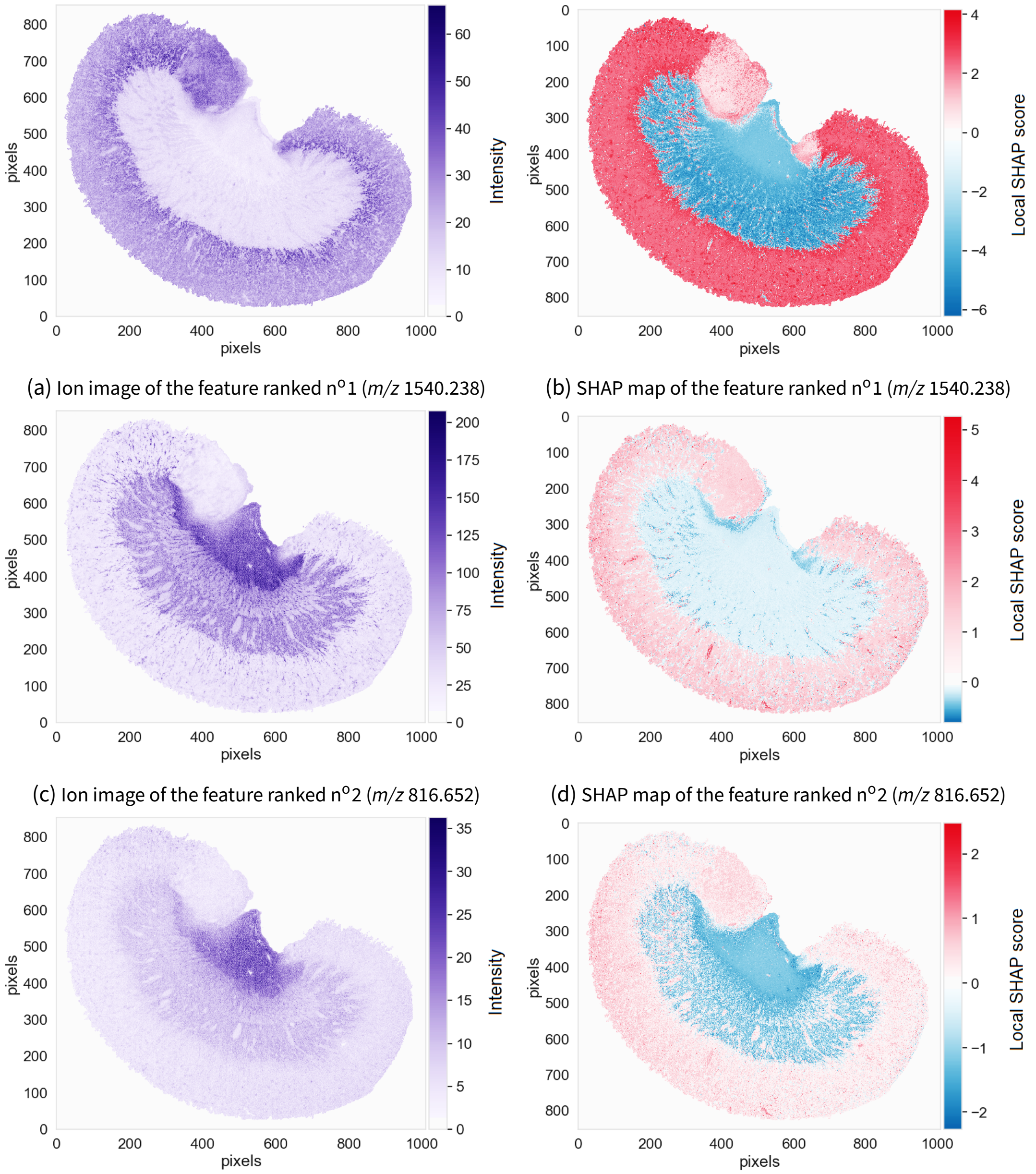

(a) Ion image of the feature ranked n°1 (*m/z* 1540.238) (b) SHAP map of the feature ranked n°1 (*m/z* 1540.238)

(c) Ion image of the feature ranked n°2 (*m/z* 816.652) (d) SHAP map of the feature ranked n°2 (*m/z* 816.652)

(e) Ion image of the feature ranked n°3 (*m/z* 1546.226) (f) SHAP map of the feature ranked n°3 (*m/z* 1546.226)

Figure S7. Three promising molecular markers for the renal cortex. The ion images (left) and SHAP maps (right) of three features (i.e. *m/z* values) with the most influence on the decision-making process of the classifier trained to recognize the rat's renal cortex are shown. The ion images plot the spatial distribution and measured intensity of each feature across the sample, and are not specifically tied to the task of recognizing the cortex. The SHAP maps plot the spatial distribution of Shapley values, or local SHAP predictive importance scores, of each feature across the sample, and provide information on where and how the feature is relevant to the task of recognizing the cortex.

References for the supplementary material:

[16] N. Verbeeck, R. M. Caprioli, and R. Van de Plas. "Unsupervised Machine Learning for Exploratory Data Analysis in Imaging Mass Spectrometry". In: *Mass Spectrometry Reviews* (Oct. 2019). DOI: 10.1002/mas.21602.

[77] I. Jolliffe. *Principal Component Analysis*. 2nd. Statistics. Springer, 2002. ISBN: 978-1-4757-1904-8.

[78] G. Liebisch et al. "Update on LIPID MAPS Classification, Nomenclature, and Shorthand Notation for MS-Derived Lipid Structures." In: *Journal of Lipid Research* 61.12 (Oct. 2020), pp. 1539–1555. ISSN: 0022-2275, 1539-7262. DOI: 10.1194/jlr.s120001025.

[79] G. Liebisch et al. "Shorthand Notation for Lipid Structures Derived from Mass Spectrometry". In: *Journal of Lipid Research* 54.6 (June 2013), pp. 1523–1530. ISSN: 1539-7262. DOI: 10.1194/jlr.M033506.
